# Responses of crop yield growth to global temperature and socioeconomic changes

**DOI:** 10.1038/s41598-017-08214-4

**Published:** 2017-08-10

**Authors:** Toshichika Iizumi, Jun Furuya, Zhihong Shen, Wonsik Kim, Masashi Okada, Shinichiro Fujimori, Tomoko Hasegawa, Motoki Nishimori

**Affiliations:** 1 Institute for Agro-Environmental Sciences, National Agriculture and Food Research Organization, Tsukuba, Japan; 2 Japan International Research Center for Agricultural Science, Tsukuba, Japan; 3 Center for Social and Environmental Systems Research, National Institute for Environmental Studies, Tsukuba, Japan

## Abstract

Although biophysical yield responses to local warming have been studied, we know little about how crop yield growth—a function of climate and technology—responds to global temperature and socioeconomic changes. Here, we present the yield growth of major crops under warming conditions from preindustrial levels as simulated by a global gridded crop model. The results revealed that global mean yields of maize and soybean will stagnate with warming even when agronomic adjustments are considered. This trend is consistent across socioeconomic assumptions. Low-income countries located at low latitudes will benefit from intensive mitigation and from associated limited warming trends (1.8 °C), thus preventing maize, soybean and wheat yield stagnation. Rice yields in these countries can improve under more aggressive warming trends. The yield growth of maize and soybean crops in high-income countries located at mid and high latitudes will stagnate, whereas that of rice and wheat will not. Our findings underpin the importance of ambitious climate mitigation targets for sustaining yield growth worldwide.

## Introduction

Given the anticipated increase in food demand in the coming decades, recent and future yield growth levels, including their patterns and rates^1–4^, are of concern to national governments and international agencies focused on agriculture and food security. Plausible estimates of climate change contributions to future yield growth have become important, because climate change is recognized as a threat to achieving sustainable global yield growth at rates needed to meet demand^5^ if no changes in trade and harvested areas are assumed.

Economic models have been used to examine yield growth patterns under various climate and socioeconomic conditions using a combination of assumptions relating to technological improvements (or *exogenous yield growth* in the economic literature)^6^ and biophysical crop model outputs as demonstrated through the Agricultural Model Intercomparison and Improvement Project (AgMIP)^7–9^. *Intrinsic productivity growth rates* ^9, 10^ serve as an example of such assumptions and are based on historical yield trends and expert opinions on the future returns of agricultural research and development (R&D). Although the combined use of assumptions and crop model outputs is methodologically valid, it has become evident that the climate change that has occurred over the past few decades has had measurable effects on the yield growth and variability of major crops^11–13^. This is despite the fact that the relative contributions of climate change to yields have until now likely been less significant than those of technological improvements and that the negative impacts of past climate change may be offset by fertilizer effects associated with elevated carbon dioxide (CO_2_) concentrations.

To this end, attempts to estimate potential future yield growth based on a biophysical crop model would benefit scientific communities in further improving estimates of adaptation costs of agriculture and food security. This is because crop models present advantages in terms of assessing yield impacts resulting from management and climate extremes and from biological limits on yields in an eco-physiologically consistent manner. Although many of the economic models included in the AgMIP consider some biophysical constraints in their outlook^14^, few have directly applied a crop model to simulate yield growth.

Furthermore, the linking of anticipated yield growth patterns with global temperature change is required to facilitate international negotiations on climate mitigation. Global temperature change is the most robust measure for climate change at the global scale, although precipitation change is relevant at the local scale. Most research on the impacts of climate change on crop production has considered yield responses to local warming by geographic region^11, 15, 16^. This is not only informative and allows for an understanding of varying yield responses and adaptation potentials across regions, but also requires the use of aggregation to infer the responses of global or country mean yields to a specific level of global temperature change, such as 1.5 °C or 2 °C (e.g., ref. 17). This shortfall can be seen in earlier work, with the notable exception of ref. 18 and, hence, research addressing yield growth responses to various warming levels is needed to fill this knowledge gap.

To the best of our knowledge, we are the first to examine the yield growth of major crops for 2010–2100 as simulated by a crop model and to associate this with global temperature changes from preindustrial levels. Mitigation levels (represented by the Representative Concentration Pathway, RCP^19^), the global climate model (GCM) and socioeconomic assumptions (represented by the Shared Socioeconomic Pathway, SSP^20^) are considered as drivers or sources of uncertainty for the yield growth outlook.

To simulate yield growth, we developed a biophysical global gridded crop model, referred to as the Crop Yield Growth Model with Assumptions on climate and socioeconomics (CYGMA), based on previous work^13^. The model includes parameterizations used to describe contributions to yields due to farmers having improved access to inputs and technologies as well as parameterizations used to describe agronomic adjustments. The former includes an increased use of improved technologies and farm field management systems. Historical patterns of the nitrogen (N) application rate were also included. The latter considers the sowing date shift and changes in a crop’s thermal requirements. The model was validated by comparing the simulated global and country mean yields for 1961–2012 with data reported by the United Nations Food and Agriculture Organization (FAO). We then conducted a 60-member ensemble yield simulation for 2001–2100 based on assumptions of N application rates, the knowledge stock of agricultural technologies derived from the SSP country gross domestic production (GDP) value, and population assumptions and bias-corrected GCM daily data for RCPs (4 RCPs × 5 GCMs × 3 SSPs).

## Results

### Validation of simulated historical yield growth

**Figure 1:**
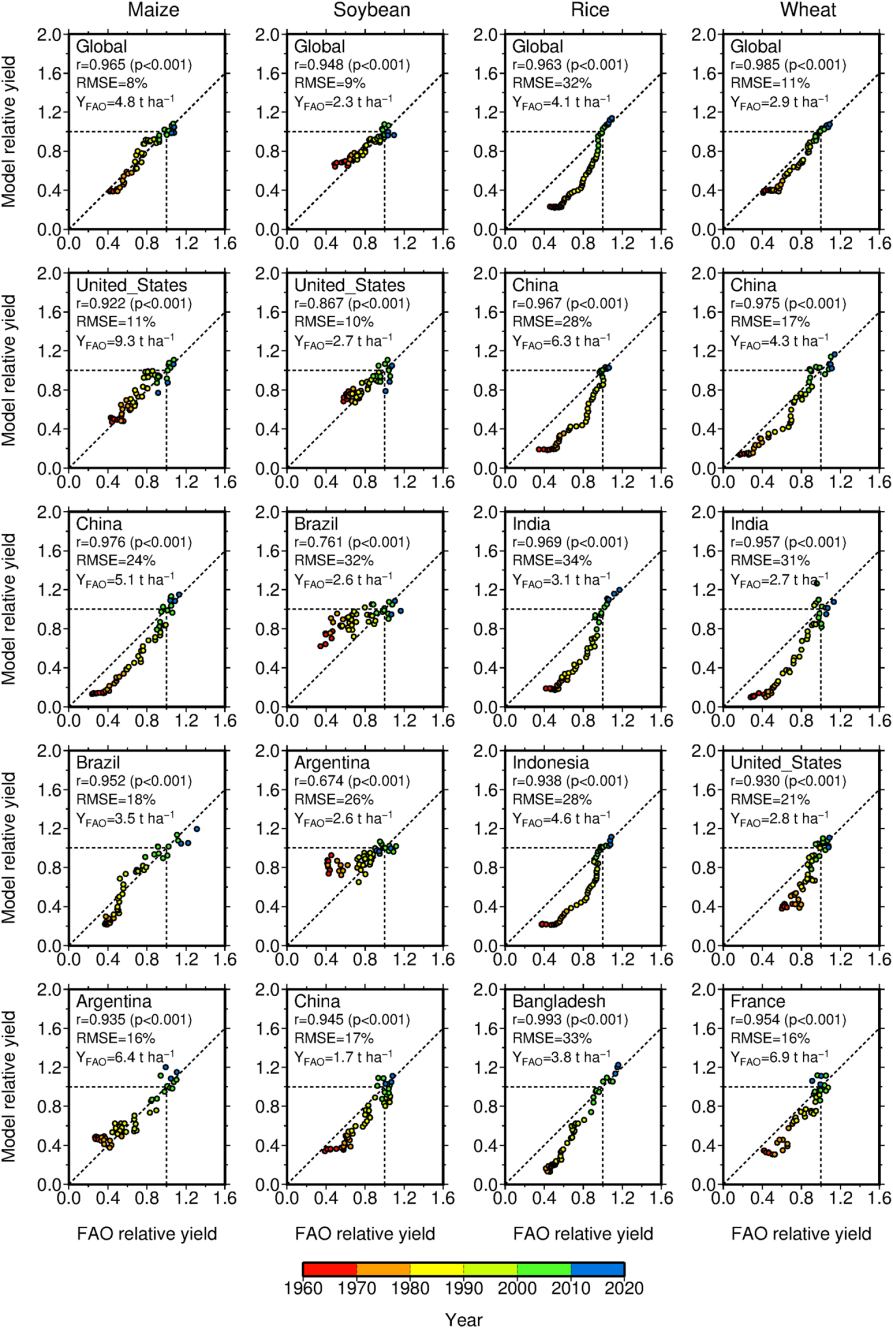
Comparisons of the 2-year running global and country mean yields of four crops for 1961–2012 between the modeled and FAO-reported data. The modeled and reported data were scaled separately to render the mean yield for 2001–2010 equal to one. The correlation (r), p-value (p), root-mean-squared error (RMSE) as a percentage of the mean reported relative yield for 1961–2012, and the mean reported absolute yield for 2001–2010 (Y_FAO_) are also presented. See Post-processing for the crop model output in Supplementary Note for more information.

The modeled global and country mean yields of four crops for 1961 to 2012 corresponded with the reported data (Fig. 1). For this comparison, the modeled data were scaled to render the mean modeled yield for 2001–2010 equal to one. The reported relative yields were calculated in the same manner. However, the modeled global mean relative yield for rice for years before 2000 showed some discrepancies with the reported data, causing the modeled rice yield growth to occur at a rate higher than the actual rate. Similar model deficiencies were found for certain crop-country combinations (e.g., rice in Indonesia and wheat in India) (Fig. 1). However, for all four crops considered here, correspondence between the modeled and reported relative yields in terms of yield growth and variability levels was found for most of the major crop-producing countries selected.

Despite the correspondence found in relative terms, we include some cautionary notes. The modeled yields showed systematic errors in absolute terms in some cases (e.g., rice in China and wheat in India) (Supplementary Fig. S1); in addition, growth rates of the modeled yield were underestimated (e.g., maize in Argentina) or overestimated (e.g., soybean in China, rice in Bangladesh and wheat in the United States (US)). Possible causes of these discrepancies are discussed below (see Discussion). Despite such discrepancies, significant correlations were calculated between the modeled and reported absolute yields for global mean yields of the four crops and for 46–84% of the crop-producing countries, with variations occurring between crops (Supplementary Fig. S2). Root-mean-square errors (RMSEs) calculated from the modeled and reported global mean absolute yields ranged from 8% (soybean) to 28% (rice) (Supplementary Figs S1 and S2). These results indicated that the model was capable of reproducing major characteristics of the reported relative growth in global and country mean yields for the past five decades.

### Anticipated global mean yield growth

**Figure 2:**
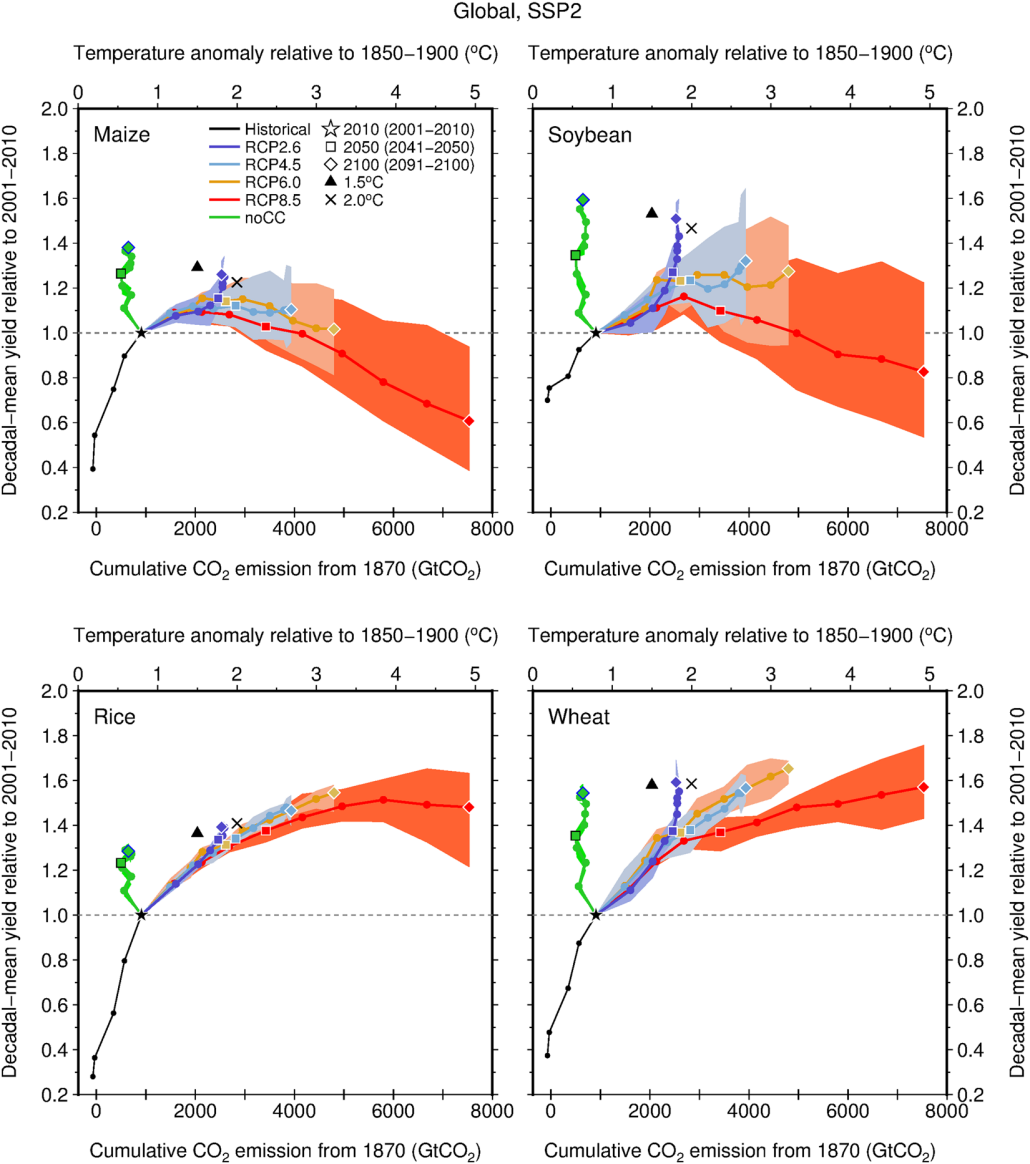
Responses of crop yield growth to global temperature changes and cumulative CO_2_ emissions from preindustrial levels under SSP2 (intermediate technological change). Decadal global mean yields of maize, soybean, rice and wheat (y-axis) are expressed as a function of cumulative total global CO_2_ emissions for 1870 (lower y-axis) or as a function of global decadal mean surface temperature anomalies relative to 1850–1900 (upper y-axis). Solid-colored lines with dots denote the ensemble mean for each RCP calculated from five GCMs. The colored-shaded area denotes the ensemble spread (from minimum to maximum) for each RCP. Data for the assumption of no climate change (noCC, five members) and SSP2 are also presented as a source of reference. Relative yields for a temperature increase of 1.5 °C were linearly interpolated from ensemble mean data derived from the noCC and RCP2.6 cases for 2100, whereas those for a temperature increase of 2 °C were based on the RCP2.6 and RCP4.5 cases for 2100.

The simulated global mean maize yield for 2100 (the average for the period 2091–2100) for the current harvested area, as denoted by the diamond symbols in Fig. 2, was found to be the highest under an assumption of no climate change followed by the yield under temperature increases of 1.8 °C (RCP2.6), 2.7 °C (RCP4.5), 3.2 °C (RCP6.0) and 4.9 °C (RCP8.5) when SSP2 (intermediate technological change) was assumed. All warming levels presented here denote the global decadal mean surface temperature anomaly relative to 1850–1900, which can be converted into cumulative CO_2_ emissions from 1870 from the relationship shown in Supplementary Fig. S3. Both quantities are directly relevant in discussing the climate mitigation targets of international negotiations. This mode of visualization is novel and more informative than the conventional mode (Supplementary Figs S4 and S5) in terms of depicting contributions to the anticipated yield growth of the CO_2_ fertilization effect and of climate change (denoted by the degree of global temperature change).

The simulated global mean maize yield for 2100 for a temperature increase of 1.8 °C and SSP2 was found to be 1.26 times greater than that for 2010 (the average for 2001–2010), and the ensemble spread across the GCMs was found to range from 1.18 to 1.32 (Fig. 2 and Supplementary Table S1). Higher (lower) levels of yield growth were always found when rapid (slow) technological changes of SSP1 (SSP3) were assumed, although differences in the simulated global mean yield for 2100 between SSP1 and SSP2 were found to be negligible for all crops regardless of the mitigation levels involved (Supplementary Fig. S5). A comparison with the no-climate-change and SSP2 case for 2100 (1.38; 1.35–1.39) showed that the global mean maize yield for a temperature increase of 1.8 °C should slightly stagnate over the long term. The yield stagnation of maize would be more severe under warming conditions (1.11: 0.96–1.29 with a 2.7 °C increase and 1.02: 0.81–1.19 with a 3.2 °C increase) and would eventually result in a net decrease in yield with a temperature increase of 4.9 °C (0.61; 0.39–0.94) relative to the yield in 2010 (Supplementary Table S1). A net decrease in the ensemble mean yield was also found for soybean, and severe levels of yield stagnation under extreme warming (4.9 °C) were found for rice despite it presenting the theoretically highest CO_2_ fertilization effect under RCP8.5. For wheat, it is anticipated that the global mean yield under extreme warming (1.57; 1.43–1.76) should not be significantly different from that of the no-climate-change case (1.54; 1.50–1.58). Importantly, the trends for the crops described above were consistent across the different socioeconomic assumptions applied (SSP1 and SSP3) (Supplementary Figs S6 and S7).

For a temperature increase of 1.8–2.4 °C by 2050 (the average for 2041–2050), a stagnation in global mean yields (square symbols in Fig. 2) was revealed for maize and soybean that became more severe with warming. By contrast, global mean yields for rice and wheat for 2050 were found to be almost the same as, or even higher than, those of the no-climate-change case, suggesting that yield stagnation will not occur for these crops in the medium term. These trends remained robust when different SSPs were used (Supplementary Figs S6 and S7).

The results described above were applied to infer the impacts of temperature increases of 1.5 °C and 2.0 °C. When SSP2 was assumed, the interpolated global mean relative yields of maize (1.29) and soybean (1.53) under a temperature increase of 1.5 °C were found to be higher than those measured under a temperature increase of 2.0 °C (1.23 for maize and 1.47 for soybean) (Fig. 2 and Supplementary Table S1). By contrast, a temperature increase of 1.5 °C resulted in lower global mean yields of rice (1.36) compared with those anticipated under a temperature increase of 2.0 °C (1.41); in addition, global mean yields of wheat under the two warming conditions were found to be comparable (1.58 for 1.5 °C and 1.59 for 2.0 °C). The different trends found under temperature increases of 1.5 °C and 2.0 °C were qualitatively the same across the SSPs (Supplementary Figs S6 and S7 and Supplementary Table S1).

### Geographic patterns of yield growth

**Figure 3:**
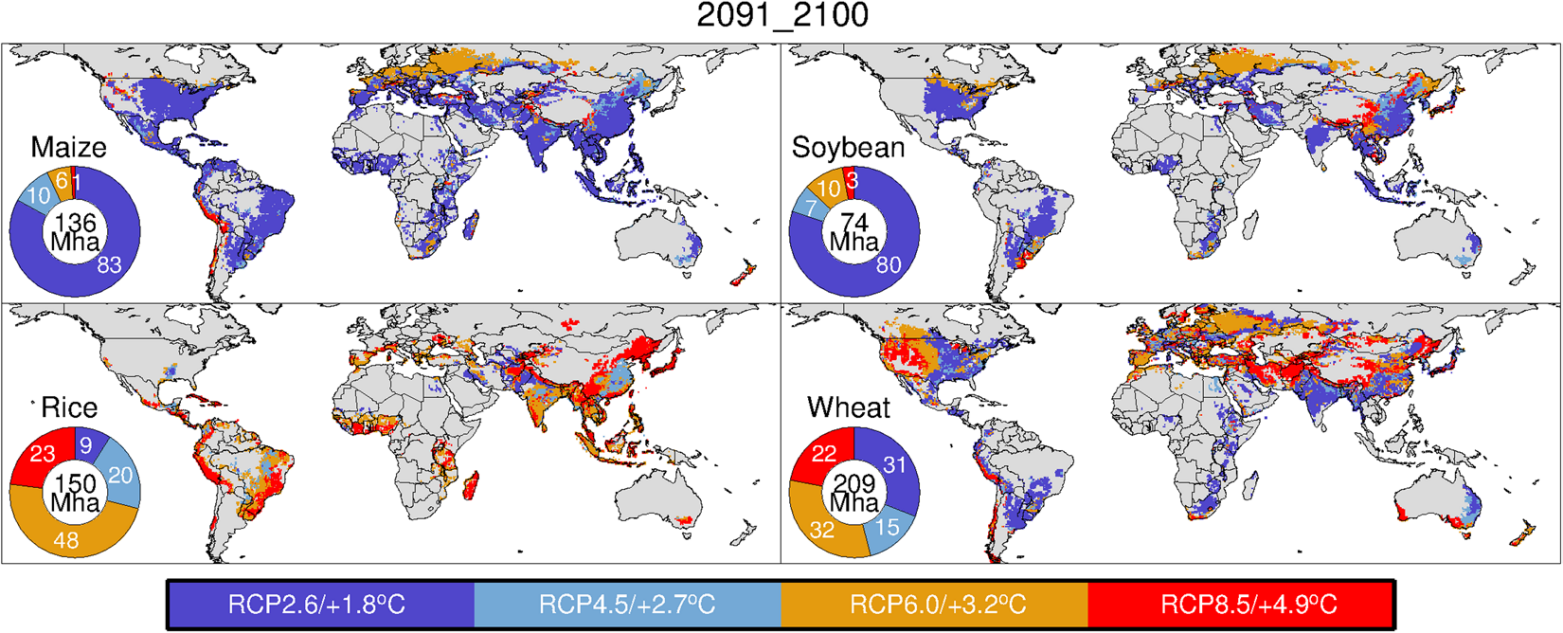
The temperature increase and corresponding mitigation level (RCP) at which the anticipated yield growth for 2100 (the average for the period 2091–2100) was found to be the highest for the four RCPs. Any warming above this level leads to yield stagnation. The pie diagrams denote percentages of harvested area under the aforementioned warming levels. All data shown in the pie diagrams are normalized to the global harvested area for 2000. The maps presented here were created from Generic Mapping Tools (GMT)^49^ version 4.5.12 (https://www.soest.hawaii.edu/gmt/) using data described in the main text.

Limited warming (1.8 °C) by 2100 would lead to yield stagnation across 9–83% of the global harvested area with variations by crop when mean results over the SSPs are used (Fig. 3). For most of the global harvested area, the simulated yields of maize (83%) and soybean (80%) for 2100 appear to stagnate. Such maize and soybean-growing regions are distributed throughout the world. By contrast, it is anticipated that the yield growth of maize and soybean in Eastern Europe, Russia and northeastern China could be maintained under higher levels of temperature increase, such as 2.7 °C and 3.2 °C. Rice yields in a substantial portion of the global harvested area in 2100 would not stagnate even under high (48%) or extreme (23%) levels of warming, whereas South Asia and the southern region of China should experience yield stagnation under less significant levels of warming (1.8 °C and 2.7 °C). For wheat, the simulated yield growth found for 32% and 22% of the global harvested area was maintained even under high or extreme levels of warming, respectively. Such regions included Eastern Europe, Russia, northeastern China, and northwestern regions of the US and Canada. However, wheat yields for 31% of the global harvested area, including South Asia, the American Midwest, the southeastern region of South America and the eastern region of Australia, are anticipated to stagnate with less significant temperature increases.

In 2050, at which point the CO_2_ fertilizer effect should theoretically be less significant than that for 2100, maize yields for 43% of the global harvested area are anticipated to stagnate with a temperature increase of 1.8 °C (Supplementary Fig. S8). For maize, the area in which simulated yields are anticipated to stagnate with temperature increases of 2.0 °C and 1.9 °C accounted for 22% and 30% of the global harvested area, respectively. These results indicate that maize yields in 95% (the sum of 43%, 22% and 30%) of the global harvested area should stagnate in the medium term with temperature increases of up to 2.0 °C. Corresponding values for soybean, rice and wheat were 94%, 38% and 68%, respectively.

### Yield growth by country income level

**Figure 4:**
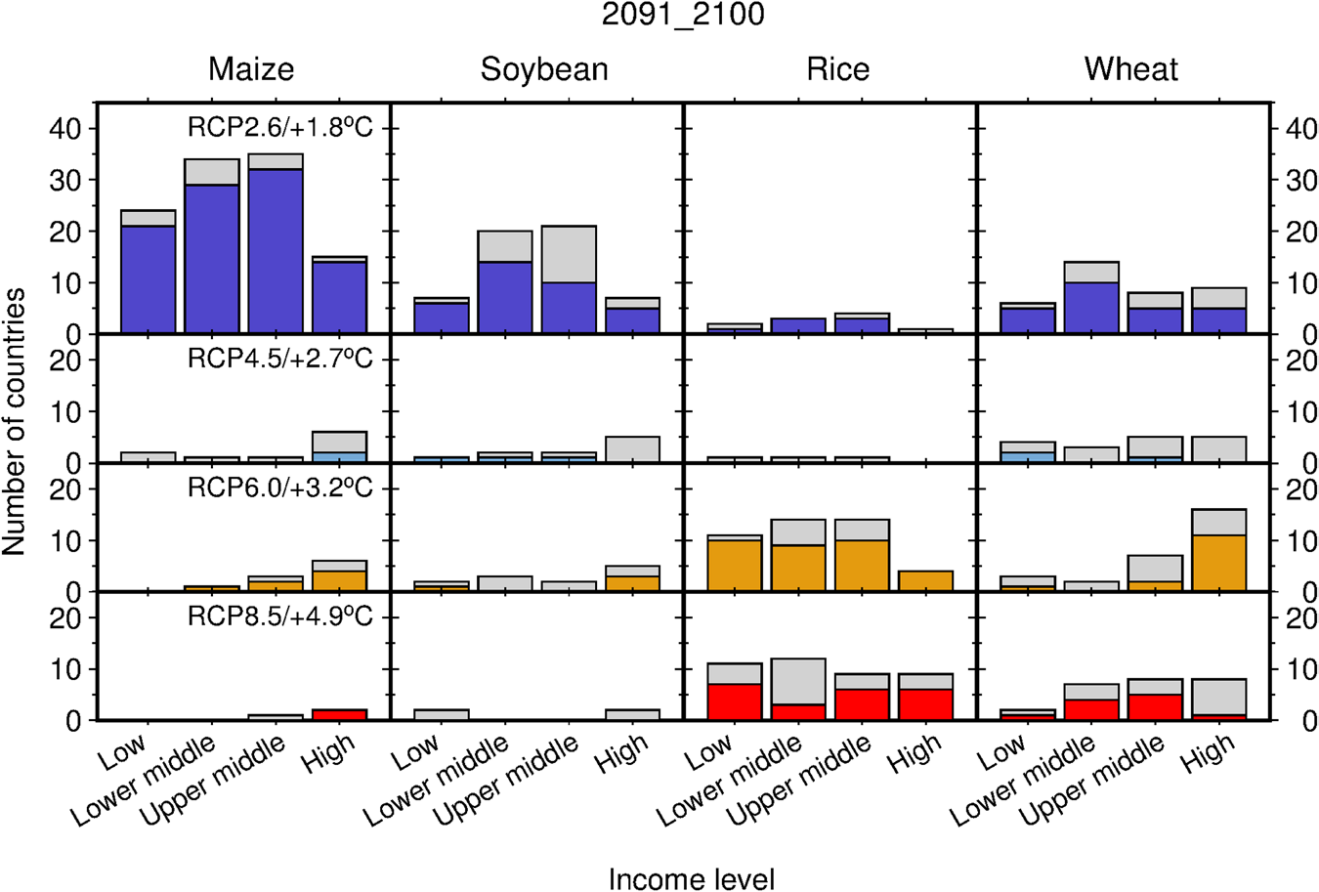
The number of countries showing the highest levels of yield growth for 2100 (the average for the period 2091–2100) by level of temperature increase and income level. The RCP corresponding to each level of temperature increase is also presented. Gray bars denote the number of countries producing a crop of interest under the aforementioned combinations of temperature increase and income. Colored bars denote the number of countries for which over 70% of the 15 ensemble members (5 GCMs × 3 SSPs) showed consistent results. The sum of bars over the four panels in a column denotes the number of countries producing a given crop.

Taking country income levels defined by the World Bank^21^ into account, the aforementioned results reveal that many low-income countries, which are often located at low latitudes, would benefit from intensive mitigation (RCP2.6) and from associated limited warming in maintaining yield growth in the long term for maize, soybean and wheat; in addition, rice yield growth could be maintained in these countries even under non-intensive mitigation (RCP6.0) and associated high levels of warming or with no mitigation (RCP8.5) under extreme warming (Fig. 4). By contrast, high-income countries, which are often located at mid and high latitudes, could benefit more from high or extreme levels of warming than from minor temperature increases (Fig. 4). This tendency was especially true for wheat and maize crops and partly true for soybean. For rice, high-income countries would benefit more from maintaining yield growth under high and extreme levels of warming than under limited levels of warming. These contrasting results between low- and high-income countries were also consistent across the medium term (Supplementary Fig. S9). Results found for other income levels (lower-middle- and upper-middle-income countries) fall between those found for low- and high-income countries regardless of the time period considered (Fig. 4 and Supplementary Fig. S9).

For all the crops examined here, higher levels of yield growth are anticipated for low- and low-middle income countries (Supplementary Fig. S10). However, higher levels of yield growth are anticipated for high-income countries, because this category includes high-latitude areas such as Northern Europe and Canada. Again, slow (rapid) technological changes assumed in SSP3 (SSP1) lead to relatively low (high) yield growth levels for all income levels. The difference in simulated yield growth levels associated with SSP could be substantial, as shown, for instance for maize in low-income countries (Supplementary Fig. S10), whereas that associated with RCP was found to be more prominent in most cases.

## Discussion

The improved correspondence reported here between modeled and reported yields in relative rather than absolute terms is consistent with other crop modeling works. This is explained by the fact that there are many facets of farm fields that reduce yields but that are not fully accounted for by the crop model^22^. Errors found in absolute terms are mainly attributed to imperfect parameterizations used in translating the total level of N fertilizer consumption into a crop-specific N application rate and in translating knowledge stock to the use of improved technologies and management systems. Information for developing countries used in these parameterizations is occasionally unreliable. This partially explains the relatively large errors in modeled absolute yields found for developing countries. Our use of country-level knowledge stock data that are coarser than the model grid size (0.5°) as model inputs also introduced errors. Our lack of explicit consideration of improved farmer access to inputs other than N fertilizer (e.g., phosphorus (P) and potash (K) fertilizers and agricultural chemicals) might have contributed to these errors. However, historical patterns of P and K fertilizer and chemical use are closely related to those of N fertilizer consumption^23–25^. Therefore, N application rates can represent producer access to inputs, and it is unlikely that a lack of information on these inputs could have significantly influenced our results. For rice and wheat, multiple cropping cycles can be completed within a year (double or triple cropping for rice in the tropics; winter and spring cropping for wheat at mid-latitudes). The model considered rainfed and irrigated conditions, but these cannot be viewed as two different cropping seasons. This may explain the relatively large differences found between the modeled and reported yields for these crops.

Our results highlight the fact that responses of global mean yield growth to global temperature change substantially differ by crop, with minor variations based on socioeconomic assumptions. The geographic distribution of crop-harvesting areas and crop-specific characteristics is central to explaining such differences. Maize is a C_4_ crop, and its CO_2_ fertilization effect saturates earlier than that of C_3_ crops (soybean, rice and wheat)^26^. As a result, the point at which the fertilization effect of C_4_ crops becomes insufficient to compensate for yield decline associated with shortened crop durations under warmer conditions occurs earlier than that for C_3_ crops. This explains why the anticipated stagnation in global mean maize yields should be more severe with warming (Fig. 2). The same principle can be used to explain why the anticipated yield growth of maize in many countries at all income levels will benefit from less warming (Fig. 4). Rice has higher optimal temperatures for growth compared with the other crops considered here (this is particularly true not only for Indica-type crops, but also for Japonica-type crops^27^), explaining why many low-income countries would be able to maintain rice yield growth even under higher levels of warming (Fig. 4). Major rice-producing regions are located in the tropics, where increases in the local temperature are always smaller than those of the global mean temperature^28^. By contrast, major soybean-producing regions are located at mid-latitudes (the US, China and Argentina), with the important exception of Brazil. Given that the increase in local temperatures at mid-latitudes is similar to the global temperature change, the results described above partially explain why the global mean soybean yield appears to be more sensitive to warming than that of rice when associated with global temperature change. Wheat is grown worldwide, and wheat yields in cooler regions could benefit from warming (e.g., Canada, the northwestern US, Northern Europe, Russia and northeastern China; Fig. 3 and Supplementary Fig. S8) as reported previously^16, 26, 29^. This partially explains why the anticipated global mean yield growth for wheat does not necessarily stagnate under warming projections. Although recent studies have reported that global mean wheat yields would decrease with warming^17, 30^, they did not consider CO_2_ fertilization effects or agronomic adjustments, which are included in this study.

Relative to high levels of warming, intensive mitigation and associated limited warming benefit many low-income countries (where rapid yield growth is needed to meet increasing national food demand) by hindering the climate-induced yield stagnation of maize, soybean and wheat (if no change in trade or harvested area is assumed). Many high-income countries, however, could maintain yield growth under higher levels of warming resulting from non-intensive or a lack of mitigation; however, high-income countries that produce maize would benefit from less warming. For rice, both low- and high-income countries would benefit more from high or extreme levels of warming than from limited warming. These trends are particularly clear in the long term, but are also consistently found for the medium term. These results corroborate the findings of ref. 26, which highlights the importance of intensive mitigation for many developing countries and suggests that related negative impacts on low-income countries in Asia may be ameliorated to some degree, given that populations in Asia rely more heavily on rice than on other crops. Nevertheless, our findings associate climate change with yield growth, which is a key measure of global food supply and of more direct relevance to global and national food security.

For each of the four crops considered here, our outlooks of global mean yields fell below anticipated demands for 2050, which are almost double those of 2005 (refs 2 and 31). Our estimates of global mean cereal yield growth for 2100 under the no-climate-change and SSP2 case relative to those for 2010 ranged from 1.28 (rice) to 1.38 (maize) to 1.54 (wheat). These values are comparable to baseline estimates derived from the Integrated Assessment Models (IAMs)^6^. We used estimates of future yield growth provided by IAMs to draw comparisons, because no yield growth data simulated from global gridded crop models are currently available. However, our estimates for maize, rice and wheat for RCP2.6 (1.26–1.59 under SSP2, Supplementary Table S1) and RCP4.5 (1.11–1.57) were lower than the anticipated levels of yield growth presented in ref. 6 (1.6 between 2005 and 2100). Only wheat showed a similar growth rate, possibly because we used the time-constant harvest area in computing global mean yields, whereas the cropland area for IAMs changes with time. In particular, SSP3 assumes a considerable expansion in cropland in developing countries where higher yield growths are anticipated (Supplementary Fig. S10), thus contributing to the observed discrepancies. These discrepancies may also be attributed to our assumptions regarding irrigation intensity levels. We assumed that the current irrigated area will remain constant without speculating on the future expansion of the irrigated area. By contrast, the IAMs consider changes in the area under irrigation. Although the contributions of our socioeconomic assumptions on global mean yields were relatively minor, they will become more significant if the harvested area changes depending on the SSP, as shown in ref. 6. Given that the approaches to yield modeling used in ref. 6 and in this study are substantively different, this discrepancy highlights opportunities to improve our understanding of assumptions on future yield growth. Such improvements will allow IAMs and global economic model intercomparison activities (e.g., AgMIP) to apply more harmonized assumptions on future yield growth patterns. However, such results must be viewed with caution, because our outlook does not necessarily represent the upper limit of future yield growth. Investments in targeted adaptation (e.g., the timely release of improved varieties specifically tailored for future climates in a particular region^32^), combined with conventional investments in improvements to plant breeding methods for higher yields, agricultural infrastructures and input-use efficiencies, will lead to higher yields than those presented here. Our outlook therefore suggests that yield growth levels are likely insufficient and that an expansion in harvested area, an increase in irrigation intensity levels and other factors (trade, dietary change and a reduction of food waste) are needed to meet future food demand.

Furthermore, our results present implications for the impacts of 1.5 °C warming, on which the Intergovernmental Panel on Climate Change (IPCC) is currently preparing a special report^33^. Our results based on the interpolated ensemble global mean yields suggest that impacts of the two warming levels (1.5 °C and 2.0 °C) could be distinguished for maize, soybean and rice, but not for wheat (Fig. 2 and Supplementary Table S1). Importantly, differences in yields between the two warming levels are comparable in magnitude to those associated with the use of different SSPs (Fig. 2 and Supplementary Figs S6 and S7). This indicates that socioeconomic assumptions constitute an important source of uncertainty when discussing differences in impacts on crop yields at temperature increases of between 1.5 °C and 2.0 °C. Although our approach is simpler than that adopted in earlier studies^18, 34^, we believe that the main differences in impacts of the two warming levels are well captured. Once the GCM outputs from the 1.5 °C stabilization experiments^33^ become available, we will be able to conduct a more detailed analysis.

This study presents some limitations. First, while uncertainties in simulated global mean yields associated with the use of different crop models are considerable^16, 26^, they are not considered here because yield growth data for other crop models are not available. Second, different maps of harvested areas generate different global mean yields^35^. Finally, as discussed in recent studies^36–38^, combining the impacts of climate on both yields and harvested areas to improve estimates of climate change contributions to yield growth will be a challenge in coming years. More detailed assumptions regarding agricultural development and local adaptation^39^ measures will also prove useful for this purpose.

To conclude, we present the first global assessment of impacts on crop yield growth associated with climate and socioeconomic changes. Our simulations based on the biophysical global gridded crop model reveal that intensive mitigation would benefit many low-income countries in terms of improving their food security levels by preventing maize, soybean and wheat yield stagnation due to climate change, although this is not the case for rice. Related impacts on global mean wheat yields with temperature increases of between 1.5 °C and 2.0 °C are not distinguishable. However, global mean maize and soybean yields with a temperature increase of 1.5 °C would stagnate less than those under a temperature increase of 2.0 °C, whereas a temperature increase of 2.0 °C is likely to benefit rice more than a temperature increase of 1.5 °C.

## Methods

### Global gridded crop model

The model used in this study was first proposed in ref. 13. The model described in this previous study was designed to calculate agro-climatic indices and to only simulate potential crop growth under optimal conditions. The model was improved for this study by including growth stress and management submodels. The spatial resolution of the model was also improved to operate at a 0.5° grid size. The improved model (referred to as the CYGMA) operates with a daily time step and simulates actual yields under irrigated and rainfed conditions.

A schematic illustration of the model is shown in Supplementary Fig. S11. Modeled crop development is expressed as a fraction of the accumulated growing-degree days (GDD) relative to crop thermal requirements. Leaf growth and senescence are computed based on the prescribed shape of the leaf area index curve and based on the fraction of the growing season considered. Yields are calculated from the level of photosynthetically active radiation intercepted by the crop canopy, the radiation-use efficiency (RUE) level, the CO_2_ fertilizer effect on RUE and the fraction of total biomass increments allocated to the harvestable component. Actual evapotranspiration is derived from the soil water balance submodel coupled with the snow cover submodel.

Five different types of stress (N deficit, heat, cold, water deficit and excess water) are considered, and the dominant type of stress for a day lowers the daily potential increment of the leaf area and yield. Soybean is a legume, and earlier crop models assume that no N deficit stress is applied to this crop. However, it is considered in our model because, in reality, N fertilizer is applied to soybean crops to prevent N deficit stress^24, 40^. Each type of stress is a function of climate, and levels of stress change as the knowledge stock increases (see The use of improved technologies and management systems in Supplementary Methods). The modeled N application rate increases and levels off according to changes in per capita GDP and per capita agricultural area (see N application rates in Supplementary Methods). These changes represent technological improvements but not planned adaptation to climate change.

Sowing dates are modeled to shift according to changes in temperature and moisture regimes. Total crop thermal requirements change with the temperature regimen, which represents the use of longer-season varieties to prevent shortened crop durations and associated yield losses. See Crop model description in Supplementary Note for more information.

### Management inputs

Information on the key inputs used—N application rates, knowledge stock, the use of improved technologies and management systems, and irrigation intensity levels—is shown in Supplementary Methods.

### Climate and other inputs

A new global and retrospective 0.5°-resolution 56-year (1958–2013) daily meteorological forcing data set, referred to as S14FD^41^, a hybrid of JRA-55 Japanese reanalysis data^42^ and gridded observations, was used for the historical period. In developing the S14FD, elevation corrections, monthly reanalysis bias corrections, and gauge type-specific wind-induced snowfall and rainfall undercatch corrections were applied to the reanalysis data.

For the future period, bias-corrected Coupled Model Intercomparison Project phase 5 (CMIP5) GCM daily data for RCPs (2.6, 4.5, 6.0 and 8.5 W m^−2^)^43^ were used. CMIP5 historical simulation data for 1961–2005 were also used after bias corrections were made. The set of GCMs used here (Supplementary Table S3) was the same as that used for the Inter-Sectoral Impact Model Intercomparison Project (ISI-MIP)^44^, although the bias correction method and reference forcing data set are different from those of ref. 44. More information on the historical and future climate data is given in ref. 41.

Other inputs include the observed annual mean CO_2_ concentration^45^; the RCP global annual mean CO_2_ concentration^19^; and the plant-extractable water capacities of soil^46^. From the model, the sowing date for 2000 was found to be similar to that of MIRCA2000 (ref. 47), but changed dynamically based on the simulated temperature and moisture regimens (see Sowing date in Supplementary Note).

### Simulation design

Three types of simulation, i.e., the historical, future and no-climate-change (noCC) runs, were conducted using the CYGMA model (Table 1). In the historical run (1960–2012), the model simulated crop yields from historical data of climate conditions, CO_2_ patterns, N application rates, knowledge stock levels and irrigation intensity levels. A 120-year (1958–1959 × 60) soil moisture spin-up was applied before the simulation was conducted.

**Table 1:** Crop model simulations conducted in this study.

Run	Climate	CO_2_	Technologies and management^1^	Irrigation intensity	Period
Historical	Historical	Historical	Historical	Historical	1960–2012
Future	Bias-corrected CMIP5 GCMs	RCP2.6, 4.5, 6.0 and 8.5	SSP1, 2 and 3	Constant 2010	2000–2100
No climate change (noCC)	Resampled historical data^2^	Constant 2010	SSP1, 2 and 3	Constant 2010	2000–2100

^1^This includes the N application rate and knowledge stock. Agronomic adjustments are considered for all runs.

^2^Climate data were randomly resampled from historical data for 1981–2010 to represent climate trends for the period. Five ensemble members were generated.

For the future run (2000–2100), we applied assumptions on climate conditions, CO_2_ patterns, N application rates, and knowledge stock levels. Model inputs used for 2000–2009 other than climatic conditions were the same as those used for the historical run, whereas the climate input for 2000–2005 was based on the bias-corrected CMIP5 historical simulation data. The irrigation intensity for 2010–2100 was kept constant at the 2010 level (Table 1). The simulated soil moisture level for December 31, 1999 obtained from the historical run was used as an initial condition.

For the noCC run (2000–2100), climate data were artificially generated. We generated a sequence of 101 yearly values by randomly sampling those for 1981 to 2010, where warming from preindustrial levels were held at the current level. We assigned the S14FD data^41^ so that they followed the sequence of sampled years, and we used these data as climate inputs for 2000–2100. This procedure was applied five times, and five ensemble members were provided for the noCC run. The CO_2_ concentration was held at the 2010 level. Inputs related to technologies and management schemes and to the initial soil moisture condition were the same as those of the future run (Table 1).

## Electronic supplementary material

Supplementary Information
