# Supplementary material for: Responses of crop yield growth to global temperature and socioeconomic changes
Source: Sci Rep. 2017 Aug 10;7:7800. doi: 10.1038/s41598-017-08214-4 (PMC5552729; doi:10.1038/s41598-017-08214-4)
Supplement: Supplementary file 1 — Supplementary Information [file 41598_2017_8214_MOESM1_ESM.pdf]

Supplementary Information

Responses of crop yield growth to global temperature and socioeconomic changes

Toshichika Iizumi, Jun Furuya, Zhihong Shen, Wonsik Kim, Masashi Okada, Shinichiro Fujimori, Tomoko Hasegawa and Motoki Nishimori

Supplementary Methods

Model inputs

N application rates. Grid-cell annual crop-specific N application rates for the period 1961–2012 were modeled by following the four steps described below using the country annual GDP and population data from the World Development Indicators[50] and the agricultural area and N fertilizer consumption data from FAOSTAT[51], as well as the grid-cell crop-specific N application rates for 2000 (ref. 23) and the crop-specific N application rates for selected countries and years[24, 40, 52]. Then, assumptions regarding the crop-specific N application rates from 2010 to 2100 were derived using the SSP country annual GDP and population data[53]. The SSP annual data were interpolated from the original data with the 5-year interval.

In the first step, we empirically associated country annual N fertilizer consumption with per capita GDP and per capita agricultural area according to ref. 54:

$$NFC_{r,t} = a \cdot \ln\left(GDP\,\mathrm{pc}_{r,t}\right) + b \cdot \ln\left(AgA\,\mathrm{pc}_{r,t}\right) + c + \varepsilon\text{, (1)}$$

where the suffixes r and t denote country and year, respectively; $NFC_{r,t}$ is the N fertilizer consumption per unit agricultural area (kg N ha^{-1} yr^{-1}); $GDP\,\mathrm{pc}_{r,t}$ is the per capita GDP (constant 2005 USD person^{-1} yr^{-1}); $AgA\,\mathrm{pc}_{r,t}$ is the per capita agricultural area (ha person^{-1} yr^{-1}); *a*, *b* and *c* are the empirical coefficients; and ε is the error term. The coefficients were determined country by country using the data for 1961–2012. These were significant in many crop-producing countries and indicated that the N fertilizer consumption increases with income growth and/or with the increase in the scarcity of per capita agricultural land in most cases (Supplementary Table S2).

In the second step, the country annual N fertilizer consumption per unit agricultural area ($NFC_{r,t}$) was converted to the N application rate for a crop (kg N ha^{-1} yr^{-1}):

$$Napp_{r,t,p} = PF_{r,t,p} \cdot NFC_{r,t}\text{, (2)}$$

where the suffix p denotes the crop. $PF_{r,t,p}$ is the time-constant partition factor, given by:

$$PF_{r,t,p} = \frac{Napp^{\mathrm{M12}}_{r,2000,p}}{NFC_{r,2000}}\text{, (3)}$$

where $Napp^{\mathrm{M12}}_{r,2000,p}$ is the country N application rate for a crop in 2000 calculated using the data of ref. 23 (kg N ha^{-1} yr^{-1}); and $NFC_{r,2000}$ is the N fertilizer consumption per unit agricultural area in 2000 (kg N ha^{-1} yr^{-1}).

In the third step, the country annual crop-specific N application rates ($Napp_{r,t,p}$) were adjusted because the N application rates obtained from ref. 23 were often higher

than those obtained from other data sources:

$$Napp'_{r,t,p} = AF_{i,p} \cdot Napp_{r,t,p}, \quad (4)$$

where the suffix i indicates the region of the world (Africa, South America, Central America, North America, Europe, Asia and Oceania); $Napp'_{r,t,p}$ is the N application rate for a crop, adjusted to be comparable with other data sources (kg N ha^{-1} yr^{-1}); and $AF_{i,p}$ is the time-constant adjusting factor. Values of this factor were derived by averaging the adjusting factors for three different data sources:

$$AF_{i,p} = \frac{1}{n_{i,p}}\left(AF_{i,p}^{\mathrm{F06}} + AF_{i,p}^{\mathrm{R12}} + AF_{i,p}^{\mathrm{H13}}\right), \quad (5)$$

where the superscripts F06, R12 and H13 indicate the data source, i.e., ref. 52 (FAO 2006), ref. 24 (Rosas 2012), and ref. 40 (Heffer 2013), respectively; $n_{i,p}$ is the number of available data sources for a region and crop. If we take the adjusting factor for ref. 24 as an example, it yields the following:

$$AF_{i,p}^{\mathrm{R12}} = \frac{1}{21}\frac{1}{m_{i,p}}\sum_{t=1990}^{2010}\sum_{r=1}^{m_{i,p}}\frac{Napp_{r,t,p}^{\mathrm{R12}}}{Napp_{r,t,p}}, \quad (6)$$

where $Napp_{r,t,p}^{\mathrm{R12}}$ is the N application rate for selected countries and crops for the period 1990–2010 reported in ref. 24; and $m_{i,p}$ is the number of countries in a region with effective data for a crop. The literature[24] reports data for maize in Brazil, China, Mexico and the US. Other adjusting factors, $AF_{i,p}^{\mathrm{F06}}$ and $AF_{i,p}^{\mathrm{H13}}$, were calculated in a similar fashion but using the data from 29 countries for 2010 or 2010/11 obtained from ref. 40 and that of 27–65 countries during the period from 1997–2004 obtained from ref. 52. For all four crops, the adjusted N application rates ($Napp'_{r,t,p}$) were truncated to not exceed a certain level (50 kg N ha^{-1} yr^{-1} for soybean and 200 kg N ha^{-1} yr^{-1} for the remaining crops), as the literature has reported the plateau of the N application rate in developed regions[55, 56]; the N application rate for maize in the US has leveled off at approximately 150 kg N ha^{-1} yr^{-1} since 1980 (ref. 57). The use of these adjusting factors ensures that the N application rates used in this study are consistent with other data (Supplementary Fig. S12).

In the final step, the adjusted country annual N application rate for a crop ($Napp'_{r,t,p}$) was translated into a grid-cell N application rate, $Napp_{g,t,p}$ (kg N ha^{-1} yr^{-1}):

$$Napp_{g,t,p} = SF_{g,p} \cdot Napp'_{r,t,p}, \quad (7)$$

where the suffix g indicates the grid cell; and $SF_{g,p}$ is the time-constant spatial disaggregation factor. Values of this factor were derived by dividing the grid-cell N application rate of 2000 ($Napp_{g,2000,p}^{\mathrm{M12}}$, kg N ha^{-1} yr^{-1}) by the country N application rate of the same year ($Napp_{r,2000,p}^{\mathrm{M12}}$):

$$SF_{g,p} = \frac{Napp_{g,2000,p}^{\mathrm{M12}}}{Napp_{r,2000,p}^{\mathrm{M12}}}. \quad (8)$$

The grid-cell N application rates were truncated at the aforementioned levels.

For the future period (2010–2100), we used the SSP country annual per capita GDP data. The agricultural area was kept constant at the 2010 level, whereas time-varying SSP population data were used to derive the country annual per capita agricultural area.

Knowledge stock of agricultural technologies. The country annual knowledge stock of the historical period was modeled using data on country annual GDP, agriculture value added and total R&D expenditure for the period 1960–2015 obtained from the World Development Indicators[50]. The future assumptions were derived using the SSP country annual GDP data[53]. The knowledge stock estimated here is not crop-specific, but in the latter procedure, it was translated into crop-specific assumptions regarding the use of improved technologies and management (see **The use of improved technologies and management systems**).

The country annual agricultural R&D expenditure, $E_{r,t}$ (constant USD 2005 yr^{-1}), was approximately calculated:

$$E_{r,t} = GDP_{r,t} \cdot \frac{AGV_{r,t}}{100} \cdot \frac{RDE_{r,t}}{100}, \quad (9)$$

where $GDP_{r,t}$ is the gross domestic production (constant 2005 USD yr^{-1}); $AGV_{r,t}$ is the agriculture value added (% of GDP); and $RDE_{r,t}$ is the total R&D expenditure (% of GDP). The comparison between the calculated agricultural R&D expenditure and reported data for the selected countries during the period 1980–2005 (ref. 58) showed good correspondence with relatively large errors in some developed countries (Supplementary Fig. S13), ensuring that our assumption regarding the historical agricultural R&D expenditure is reasonable.

The literature[59] employed a knowledge stock accumulation equation based on the conventional capital accumulation equation. Another study[60] also used this equation to take the duration of research into account. Based on these studies, the country knowledge stock for a year, $R_{r,t}$ (constant 2005 USD yr^{-1}), was calculated:

$$R_{r,t} = E_{r,t-6} + (1-\delta)R_{r,t-1}, \quad (10)$$

where δ is the obsolescence rate of technological knowledge; and the lag time between research and technology adoption by farmers was set at six years. The value of δ was set at 0.1, as in ref. 60. Although lag time may vary by country and over time, other works use a similar order of lag time from seven to nine years[61, 62]. Caution is necessary in the use of a constant lag time and obsolescence rate for long-term analysis, as it becomes increasingly difficult to find new ways to increase yields in regions where yields are already high[61]; in the US, there are concerns that returns of agricultural R&D have been declining[63]; in our modeling, the contributions from international agricultural research organizations to the knowledge stock in developing countries are found to be lacking.

In the future period, the SSP country annual GDP data were used. Agriculture value added and total R&D expenditure were kept constant at their mean 2006–2010 levels, although this assumption may be somewhat unrealistic because the GDP share of agriculture has decreased and that of total R&D expenditure has increased with economic growth[50]. Additionally, agricultural R&D expenditure has continued to increase at different rates in high- and middle-income countries[64].

The use of improved technologies and management systems. The knowledge stock was translated into the use of improved technologies and management systems on farm fields. We assumed that improved varieties are central to new technologies but did not exclude other technologies, as the literature reports that improved varieties explain one-fourth to half of the past yield growth, and improved management, including the increased use of synthetic fertilizers, irrigation, chemicals and machinery, and improved input-use efficiency are the reasons for the remaining portion of the yield growth[65, 66].

In the crop model, we associated the knowledge stock with the curvature factor value that represents a crop's tolerance to stresses (heat, cold, water deficit and water excess) to represent the increased use of improved technologies and management systems with economic growth. Nitrogen deficit stress was also associated with the knowledge stock to represent the reported increase in N-use efficiency[55, 56, 67]. The form of the stress function (equations (27)–(31)) and the method of calibration were the same across the stress types and crops (see also **Tolerance to stresses** in **Supplementary Note**); hence, we used the heat stress as an example for explanatory purposes.

The heat stress factor for the *i*-th day after sowing, $\gamma_{\mathrm{heat},\,i}$, is computed (equation (28)):

$$\gamma_{\mathrm{heat},\,i} = \begin{cases} 1 & T_i \le T_{\mathrm{o}} \\ \exp\left[\dfrac{-\alpha_{\mathrm{heat}}\left(T_{\mathrm{o}} - T_i\right)^2}{\left(T_{\mathrm{u}} - T_i\right)^2}\right] & T_{\mathrm{o}} < T_i < T_{\mathrm{u}} \\ 0 & T_{\mathrm{u}} \le T_i \end{cases}, \quad (11)$$

where α_{heat} is the curvature factor for heat stress; T_{i} is the daily mean temperature (°C); and T_{o} and T_{u} are the crop-specific optimal and maximum temperatures for growth (°C), respectively. The values of the stress factor ($\gamma_{\mathrm{heat},\,i}$) range from 0 under the severely stressed condition to 1 under the optimal condition.

Here, we assumed that the mean heat stress factor of the crop duration ($\bar{\gamma}_{\mathrm{heat},\,t}$) can be approximated based on the mean temperature during the same period ($\bar{T}_t$, °C):

$$\bar{\gamma}_{\mathrm{heat}\,t} = \exp\left[\frac{-\alpha_{\mathrm{heat}}\left(T_{\mathrm{o}} - \bar{T}_t\right)^2}{\left(T_{\mathrm{u}} - \bar{T}_t\right)^2}\right] \quad T_{\mathrm{o}} < \bar{T}_t < T_{\mathrm{u}}. \quad (12)$$

We also assumed that if the yield gap in a region is dominantly caused by a single stress type, then the mean stress factor due to the most dominant stress type should explain the yield gap. The yield gap used here was defined as the ratio between actual and attainable yields (Y_{a} and Y_{p}, t ha^{-1}, respectively). Using these assumptions, equation (12) is rewritten for a region where heat stress is dominant:

$$\ln\left(\bar{\gamma}_{\mathrm{heat}\,t}\right) = \ln\left(\frac{Y_{\mathrm{a}}}{Y_{\mathrm{p}}}\right) = \frac{-\alpha_{\mathrm{heat}}\left(T_{\mathrm{o}} - \bar{T}_t\right)^2}{\left(T_{\mathrm{u}} - \bar{T}_t\right)^2} \quad T_{\mathrm{o}} < \bar{T}_t < T_{\mathrm{u}}. \quad (12')$$

Then, the value of the curvature factor for heat stress (α_{heat}) is algebraically obtained:

$$\alpha_{\mathrm{heat}} = \frac{-\ln\left(\dfrac{Y_{\mathrm{a}}}{Y_{\mathrm{p}}}\right)\left(T_{\mathrm{u}} - \bar{T}_t\right)^2}{\left(T_{\mathrm{o}} - \bar{T}_t\right)^2} \quad T_{\mathrm{o}} < \bar{T}_t < T_{\mathrm{u}}. \quad (12'')$$

We associated grid-cell values of the curvature factor for heat stress with the knowledge

stock ($R_{r,t}/10^9$, billion constant 2005 USD yr^{-1}), assuming a linear relationship:

$$\alpha_{\text{heat}\,g,t} = f_{\text{heat}} \cdot \left(\frac{R_{r,t}}{10^9}\right) + g_{\text{heat}}, \quad (13)$$

where f_{heat} and g_{heat} are the empirical coefficients. The yield gap data are based on the actual yields[68] and attainable yields determined by identifying high-yielding areas within similar climate zones[23].

To determine the coefficient values of heat stress (f_{heat} and g_{heat}) for a crop, we specified regions where the heat stress is dominant by using the following steps:

i) Stress factors were calculated by the crop model using the default curvature factors values (Supplementary Table S4) and no adjustment for the knowledge stock;
ii) Grid cells in which the modeled N deficit stress was minor were specified. The threshold for this was the 80th percentile value of the mean modeled N deficit stress factor for the period 1996–2005 over the global harvested area of a crop;
iii) Grid cells in which the modeled cold stress was minor were specified using a certain threshold for cold stress (80th percentile), as was done for the N deficit stress. This step was repeated for the remaining stress types (water deficit and water excess);
iv) The grid cells specified in step ii) and iii) were combined to select the grid cells in which all stresses other than the heat stress were minor;
v) Values of the curvature factor for the heat stress in the selected grid cells were calculated according to equation (12”) and associated with the mean knowledge stock values for the period 1996–2005 (Supplementary Fig. S14); and
vi) Steps ii) to v) were repeated for the remaining stress types to determine their coefficient values.

For all crops and stress types examined here, negative slope values consistently emerged (Supplementary Fig. S14), indicating that the curvature factor value decreases (i.e., the crop’s tolerance increases) as the knowledge stock increases. These results are consistent with the fact that improved varieties often accompany higher tolerance to suboptimal conditions[66, 69, 70]. Although the values of the coefficients were not always significant because of the inconsistent spatial resolution between the two variables (i.e., grid-cell curvature factor versus country knowledge stock), we used the relationships addressed here in the crop model (Supplementary Fig. S15).

Irrigation intensity. The extent of crop-specific irrigated and rainfed areas was obtained from the Monthly Irrigated and Rainfed Crop Area around the year 2000 dataset referred to as MIRCA2000 (ref. 47). In addition, the annual growth rates of the area equipped for irrigation for 1900–2005 were obtained from the global historical irrigation dataset referred to as HID[71]. Although HID offers grid-cell estimates of the annual growth rate of an irrigated area, no crop-specific information is available. We therefore assumed that the annual growth rate of the irrigation intensity (the ratio of irrigated area to harvested area) was the same across the crops, although this assumption may be unrealistic for a certain location-crop combination.

The growth rates of irrigation intensity for 2006–2012 were extrapolated using a linear regression based on the data for the period 1998–2005. We assumed that the

harvested area derived from MIRCA2000 did not change with time, but the irrigation intensity in the historical period did change with time according to HID. Thus, this study partially accounted for the contribution to historical yield growth from the increased irrigation intensity. In the future period, the irrigation intensity was kept constant at the 2010 level.

Supplementary Note

Crop model description

A schematic overview of the CYGMA model is shown in Supplementary Fig. S11. The following sections describe the modeled processes of development, growth, yield formation, stresses and soil moisture in detail.

Development. The crop total GDD requirement from sowing to harvest, GDD_{c} (°C days), is calculated using the mean annual GDD with a base temperature of 0 °C, GDD_{a} (°C days)[72]:

$$GDD_{\mathrm{c},t} = a \cdot \left(1 - \exp\left[\frac{-GDD_{\mathrm{a},t}}{b}\right]\right). \quad (14)$$

For each year t, the mean annual GDD is calculated using the daily mean temperature over the last 10 years (t-10:t-1). a and b are the empirical coefficients (Supplementary Table S4).

The development of a crop for the i-th day after sowing, fr_{GDD} (0=sowing and 1=harvest), is expressed as a fraction of the crop growing season:

$$fr_{GDD} = \frac{\sum_{i=1}^{i} \max\left(0, \min\left[T_i, T_{\mathrm{u}}\right] - T_{\mathrm{b}}\right)}{GDD_{\mathrm{crop}}}, \quad (15)$$

where T is the daily mean temperature (°C); and T_{u} and T_{b} are the maximum and base temperatures for growth (°C), respectively (Supplementary Table S4). In this model, we only consider spring wheat (i.e., the vernalization in winter wheat is not considered).

Growth. The model simulates the daily growth of leaves and canopy height. However, the growth of roots is not considered, and the time-constant root depth is used in the model. The leaf area index is calculated based on ref. 73. Before leaf senescence becomes the dominant growth process:

$$LAI_i = LAI_{i-1} + \Delta LAI_i \qquad fr_{GDD,i} < fr_{GDD,\mathrm{sen}}, \quad (16)$$

where ΔLAI is the increment of the leaf area index for day i (m^2 m^{-2}); LAI_i and LAI_{i-1} are the leaf area indices for days i and i-1, respectively (m^2 m^{-2}); and $fr_{GDD,\mathrm{sen}}$ is the fraction of the growing season at which senescence becomes the dominant growth process (Supplementary Table S4). The daily increment of the leaf area index is given as follows:

$$\Delta LAI_i = \left(fr_{LAI\max,i} - fr_{LAI\max,i-1}\right) \cdot LAI_{\max} \cdot \left\{1 - \exp\left(5 \cdot \left[LAI_{i-1} - LAI_{\max}\right]\right)\right\} \cdot \gamma_i, \quad (17)$$

where $fr_{LAI\max,i}$ and $fr_{LAI\max,i-1}$ are the fractions of the maximum leaf area index under the optimal condition for days i and i-1, respectively; $LAI_{\max}$ is the maximum leaf area index (m^2 m^{-2}, Supplementary Table S4); and γ is the most dominant stress for day i (see

Stresses in **Supplementary Note**). The fraction of the maximum leaf area index, $fr_{LAI\mathrm{max}}$, is related to the fraction of the crop growing season:

$$fr_{LAI\mathrm{max},i} = \frac{fr_{GDD,i}}{fr_{GDD,i} + \exp(l_1 - l_2 \cdot fr_{GDD,i})}, \quad (18)$$

where l_1 and l_2 are the empirical coefficients (Supplementary Table S4). Once leaf senescence becomes the dominant growth process, the leaf area index decreases as the fraction of the growing season increases:

$$LAI_i = LAI'_{\mathrm{max}} \cdot \frac{(1 - fr_{GDD,i})}{(1 - fr_{GDD,\mathrm{sen}})} \quad fr_{GDD,i} \geq fr_{GDD,\mathrm{sen}}, \quad (19)$$

where LAI'_{max} is the maximum leaf area index achieved in a growing season (m^2 m^{-2}).

The canopy height of a crop is calculated[73]:

$$h_i = h_{\mathrm{max}} \cdot \sqrt{fr_{LAI\mathrm{max},i}}, \quad (20)$$

where h is the canopy height for day i (m); h_{max} is the maximum canopy height of a crop (m, Supplementary Table S4); and $fr_{LAI\mathrm{max}}$ is the maximum leaf area index corresponding to a given fraction of the growing season. Once the maximum canopy height is reached, the canopy height remains constant until harvest.

Yield formation. Once the crop reaches anthesis, the model starts to calculate the amount of the harvestable part of a crop (yield):

$$yld_i = yld_{i-1} + \Delta yld_i, \quad (21)$$

where yld_i and yld_{i-1} are the yields for days i and i-1 (t ha^{-1}), respectively; and Δyld_i is the increment of yield for day i (t ha^{-1}). The daily increment of yield is calculated:

$$\Delta yld_i = \frac{\Delta bio_i}{1000} \cdot fr_{\Delta bio,i} \cdot \gamma_i, \quad (22)$$

where $\Delta bio/1000$ is the increase in crop total biomass for day i (t ha^{-1}); $fr_{\Delta bio}$ is the fraction of the increment of the total biomass allocated to the harvestable part of a crop; and γ is the most dominant stress for day i. The partition fraction for the harvested part is calculated[74]:

$$fr_{\Delta bio,i} = \begin{cases} \dfrac{fr_{GDD,i}}{fr_{GDD,i} + \exp(p_1 - p_2 \cdot fr_{GDD,i})} & fr_{GDD,i} \geq fr_{GDD,\mathrm{ant}} \\ 0 & fr_{GDD,i} < fr_{GDD,\mathrm{ant}} \end{cases}, \quad (23)$$

where $fr_{GDD,\mathrm{ant}}$ is the fraction of the growing season at which anthesis occurs; and p_1 and p_2 are empirical coefficients (Supplementary Table S4). The increment of the potential total crop biomass is calculated using the radiation-use efficiency for year t, RUE ((kg ha^{-1}) (MJ m^{-2} d^{-1})$^{-1}$), and the intercepted photosynthetically active radiation for day i, PAR (MJ m^{-2} d^{-1}):

$$\Delta bio_i = RUE_t \cdot PAR_i. \quad (24)$$

The intercepted photosynthetically active radiation is calculated[73]:

$$PAR_i = 0.5 \cdot SR_i \cdot (1 - \exp[-k \cdot LAI_i]), \quad (25)$$

where SR is the downward shortwave radiation (MJ m^{-2} d^{-1}); and k is the light extinction coefficient (Supplementary Table S4). The relationship between the annual mean CO_2 concentration for year t, CO_2 (ppm), and radiation-use efficiency is modeled[73]:

$$RUE_t = \frac{100 \cdot CO_{2,t}}{CO_{2,t} + \exp(r_1 - r_2 \cdot CO_{2,t})}, \quad (26)$$

where r_1 and r_2 are empirical coefficients (Supplementary Table S4).

Stresses. The model accounts for five different stress types: N deficit, heat, cold, water deficit and water excess. These stress factors vary between 0 under the severely stressed condition and 1 under the optimal condition. The heat, cold, water deficit and water excess stresses are calculated day by day, whereas the N deficit stress is computed on an annual basis due to the lack of N application rate data at a finer temporal resolution.

The N deficit stress factor for year t, γ_{Ndef}, is calculated:

$$\gamma_{\text{Ndef},t} = \begin{cases} 0 & Napp_t \le Napp_{\min} \\ \exp\left[\dfrac{-\alpha_{\text{Ndef}}\left(Napp_{\text{o}} - Napp_t\right)^2}{\left(Napp_{\min} - Napp_t\right)^2}\right] & Napp_{\min} < Napp_t < Napp_{\text{o}} \\ 1 & Napp_{\text{o}} \le Napp_t \end{cases}, \quad (27)$$

where $Napp$ is the N application rate (kg N ha^{-1} year^{-1}); $Napp_{\text{o}}$ is the optimal N application rate; $Napp_{\min}$ is the shape parameter used to determine the minimum N deficit stress; and α_{Ndef} is the curvature factor for the N deficit stress that represents the crop's tolerance to the N deficit stress (Supplementary Table S4). The N deficit stress changes with the annual update of the N application rate, but it is constant across days within a growing season. A similar simplification is found in ref. 75.

The heat stress factor, γ_{Thigh}, is a function of the daily mean temperature (T, °C), the optimal temperature (T_{o}, °C), the maximum temperature for growth (T_{u}, °C) and the curvature factor for heat stress (α_{heat}):

$$\gamma_{\text{Thigh},i} = \begin{cases} 1 & T_i \le T_{\text{o}} \\ \exp\left[\dfrac{-\alpha_{\text{heat}}\left(T_{\text{o}} - T_i\right)^2}{\left(T_{\text{u}} - T_i\right)^2}\right] & T_{\text{o}} < T_i < T_{\text{u}} \\ 0 & T_{\text{u}} \le T_i \end{cases}. \quad (28)$$

The relationship used to calculate the cold stress factor, γ_{Tlow}, is as follows:

$$\gamma_{\text{Tlow},i} = \begin{cases} 0 & T_i \le T_{\text{b}} \\ \exp\left[\dfrac{-\alpha_{\text{cold}}\left(T_{\text{o}} - T_i\right)^2}{\left(T_{\text{b}} - T_i\right)^2}\right] & T_{\text{b}} < T_i < T_{\text{o}} \\ 1 & T_{\text{o}} \le T_i \end{cases}, \quad (29)$$

where T_{b} is the base temperature (°C) and α_{cold} is the curvature factor for cold stress. The form of the function used here is the same as that used in ref. 73; however, the heat and cold stresses are separately modeled in this study.

The water deficit stress factor, γ_{Wdef}, is modeled using an actual-potential evapotranspiration ratio, $E_{\text{a}}/E_{\text{p}}$, and the curvature factor for the water deficit stress, α_{Wdef}:

$$\gamma_{\text{Wdef},i} = \begin{cases} 0 & E_{\text{a},i}/E_{\text{p},i} = 0 \\ \exp\left[\dfrac{-\alpha_{\text{Wdef}}\left(1 - E_{\text{a},i}/E_{\text{p},i}\right)^2}{\left(E_{\text{a},i}/E_{\text{p},i}\right)^2}\right] & E_{\text{a},i}/E_{\text{p},i} > 0 \end{cases}. \quad (30)$$

Under the irrigated condition, water is applied on day i+1 to increase the root-zone soil moisture to 80% of the plant-extractable soil water capacity when the water deficit stress factor for day i is below 0.9.

The relationship used to calculate the water excess stress factor, γ_{Wexs}, is as follows:

$$\gamma_{\mathrm{Wexs},i} = \begin{cases} 1 & SW_i/SW_{\max} \le 0.9 \\ \exp\left[\dfrac{-\alpha_{\mathrm{Wexs}}\left(0.9 - SW_i/SW_{\max}\right)^2}{\left(1 - SW_i/SW_{\max}\right)^2}\right] & 0.9 < SW_i/SW_{\max} < 1 \\ 0 & SW_i/SW_{\max} = 1 \end{cases}, \quad (31)$$

where SW is the root-zone soil moisture for day i (mm); $SW_{\max}$ is the plant-extractable water capacity of the soil (mm); and α_{Wexs} is the curvature factor for the water excess stress. The water excess stress factor is calculated after three consecutive days of continued soil saturation (soil moisture exceeds 90% of the plant-extractable water capacity of the soil), as in ref. 76. The form of the function for the water deficit and excess stresses is based on ref. 77.

The calculated stress factors due to the N deficit, heat, cold, water deficit and water excess are adjusted for the knowledge stock to account for the use of improved technologies and management systems in developed countries. Taking the water deficit stress as an example, the adjustment is conducted through the modification of the curvature factor value:

$$\alpha_{\mathrm{Wdef}\ g,t} = f_{\mathrm{Wdef}} \cdot \left(\frac{R_{r,t}}{10^9}\right) + g_{\mathrm{Wdef}}, \quad (32)$$

where $\alpha_{\mathrm{Wdef},g,t}$ is the curvature factor for the water deficit stress for a grid cell and year; $R_{r,t}/10^9$ is the knowledge stock of agricultural technologies (billion constant 2005 USD yr^{-1}); and f_{Wdef} and g_{Wdef} are the empirical coefficients (Supplementary Table S4). The calculated curvature factor value is truncated to not exceed the lower and upper bounds (Supplementary Table S4), ensuring the biological limits of crop growth (Supplementary Fig. S15). Given the form of parameterization, the crop's tolerance to the water deficit increases as the knowledge stock increases. The stress factors associated with the N deficit, heat, cold and water excess are adjusted in a similar manner. The most dominant stress for day i, γ, is then selected to decrease the daily potential increment of leaf area and yield:

$$\gamma_i = \min\left(\gamma_{\mathrm{Ndef},t}, \gamma_{\mathrm{heat},i}, \gamma_{\mathrm{cold},i}, \gamma_{\mathrm{Wdef},i}, \gamma_{\mathrm{Wexs},i}\right). \quad (33)$$

Soil moisture. The root-zone soil moisture is calculated based on the water balance equation used in ref. 13, which takes rainfall, snow melt, evapotranspiration, runoff, ground water loss through deep percolation and plant-extractable water capacity of the soil into account. The rainfall-snowfall separation and snow accumulation, melt and sublimation are calculated using the daily maximum and minimum temperatures and precipitation as the inputs of the snow cover submodel[78]. Surface runoff, subsurface runoff and ground water loss are calculated based on ref. 79 after revising this submodel to operate with a daily step instead of the original monthly step. Potential evapotranspiration is calculated by using a variant of the Penman-Monteith equation[73]. Actual evapotranspiration is determined by comparing the root-zone soil moisture and potential evapotranspiration. The plant-extractable water capacity of the soil data

estimated using the soil texture, soil organic content, and plant-root (or soil profile) depth were obtained from ref. 46.

Calibration

Crop total GDD requirement. We addressed the relationships between the mean annual GDD for the period 1996–2005 and the crop total GDD requirement in 2000, as in ref. 72. Sowing and harvesting dates were obtained from MIRCA2000 (ref. 47) with the assumption that the sowing (harvesting) date was the middle day of the reported sowing (harvesting) month. The daily mean temperature data obtained from S14FD[41] were used to calculate the GDD. The crop calendar information provided by MIRCA2000 (ref. 47) is crude (a single value was often assigned for all of the cropland in a country). Therefore, for the irrigated cropping system, we selected a single grid cell with the most extensive irrigated area of a crop for each country. The same calculation was conducted for the rainfed cropping system. Using the grid-cell sowing and harvesting dates and temperature data, the relationship was specified for each crop and cropping system (Supplementary Fig. S16).

Sowing date. The modeled sowing date changes on an annual basis with any update of the climate bin because refs. 72 and 80 suggested the importance of the temperature and moisture regimes in determining the sowing date. For the calibration, first, we calculated the climate bin using the 10-year mean annual GDD and aridity index (annual precipitation divided by annual potential evapotranspiration of reference crop, P/PET[81]) for the period 1996–2005 (Supplementary Fig. S17). Second, the mean sowing date was calculated for each climate bin and cropping system (irrigated or rainfed) using the reported sowing date in 2000. Third, for the irrigated cropping system, we selected a single grid cell with the most extensive irrigated area of a crop for each country (as presented in **Crop total GDD requirement** in **Supplementary Note**) and recorded the corresponding climate bin. Fourth, the mean sowing date across countries was calculated for each climate bin after separating the data from around the world into data from the northern and the southern hemisphere. Finally, if the sowing date for a climate bin was lacking, it was extrapolated using the data in the four neighboring climate bins (Supplementary Fig. S18). The same calculation was performed for the rainfed cropping system. This calibration led to reasonable sowing dates similar to but more spatially detailed than those of MIRCA2000 (ref. 47) (Supplementary Fig. S19).

Tolerance to stresses. Each crop's tolerance to stresses is modeled to change as the knowledge stock increases. The relationship between the tolerance (represented by the curvature factor values in equations (27)–(31)) and knowledge stock was specified for each crop and stress type using the spatial data in 2000 (Supplementary Fig. S14). This indicates that we applied the relationship addressed using the difference across the developed and developing countries in 2000 to our outlook. We assumed that the developing countries in 2000 grew their economies according to the socioeconomic assumption and improved the technologies and management used in their crop production systems to be more tolerant, as seen in developed countries in 2000. More details on the calibration are presented in **The use of improved technologies and management systems** in **Supplementary Methods**.

Limitations of the crop model

The model used in this study does not necessarily represent all characteristics of plant breeding and improvements in agronomic management during the last few decades. The increased N fertilizer input is largely due to the characteristics of modern semi-dwarf varieties, which can respond better to high N input than traditional tall varieties to become liable to load and to contribute to yield growth[66]. We considered the increased N fertilizer input but not other important characteristics of modern semi-dwarf varieties, such as increased harvest index and shortened plant height[66]. Additionally, the contributions of farm machinery to weed and pest control, timeliness of planting and harvest efficiency to yield growth[82] are lacking in the model.

We considered the increased crop tolerance. This is reasonable because new varieties often accompany higher tolerances to suboptimal conditions[66, 69, 70]. However, the consideration of some changes in agronomic management that accompany new varieties is lacking. For instance, during the last 50 years, plant density in the American Midwest has increased because of biotechnology-driven increase in crop tolerance to pest, disease, drought, and herbicide and the improvement in soil management[70, 83].

Furthermore, the model does not fully consider changes in some traits that, in reality, accompany plant breeding for higher yield. If maize is taken as the example, such traits include ear height (there is a reducing trend in ear height), leaf angle (a more upright leaf orientation), staygreen (delayed leaf senescence), grain-filling period (newer hybrids have a longer period of grain fill with faster final dry-down rate), and kernel weight (increased weight per kernel)[70]. Additionally, plant breeding offers efficient light capture and harvest indices close to their theoretical maxima, leaving the efficiency of conversion of the intercepted light into biomass as the only remaining major prospect for improving yield potential in the coming decades[84]; however, this view is not incorporated into our model and outlook.

Post-processing of crop model output

The model outputs from the historical run were compared with the reported yields[51]. We calculated the modeled global and country mean yields by combining the simulated grid-cell yields of irrigated and rainfed cropping systems. In the model, the harvested year could vary by cropping system because a different cropping system obtained from MIRCA2000 (ref. 47) sometimes indicated a different cropping season. This is the case, for example, in the tropics, where monsoons play a critical role in determining water availability. For a consistent comparison, the following procedure was used:

i) We averaged over the simulated grid-cell yields produced using the irrigated and rainfed cropping systems, with both being harvested in the same year in the model, using the irrigated and rainfed areas in 2000 (ref. 47) as the weights;
ii) The calculated grid-cell cropping-system mean yields were averaged using the grid-cell harvested area in 2000 (ref. 68) as the weight to obtain the country mean yield;
iii) Global mean modeled and reported yields were calculated from the country mean yields using the FAO country harvested area[51] as the weight; and
iv) We averaged the modeled and reported global and country mean yields over two consecutive years (t-1 and t) to obtain the 2-year moving averaged data.

As noted in ref. 85, the FAO's definitions state that "yield data are calculated by dividing production by harvested area" and that "when the production data available

refers to a production period falling into two successive calendar years and it is not possible to allocate the relative production to each of them, it is usual to refer production data to that year into which the bulk of the production falls". Therefore, a year-to-year comparison between the reported and modeled country mean yields is difficult to justify, except for countries where a single annual harvest is dominant. The 2-year moving averaging ameliorates the errors to some extent. For the future and noCC runs, however, the 2-year moving average was not used because decadal mean modeled yields were analyzed. The harvested area used as the weights was kept constant at the 2010 level for the future and noCC runs.

Global temperature change and cumulative CO_2 emission

We reconstructed the relationship between the cumulative total global CO_2 emission from 1870 and the global decadal mean combined land and ocean surface temperature anomaly relative to preindustrial levels (1850–1900) (Supplementary Fig. S3). Cumulative CO_2 emission was computed using the reported emission estimates from fossil-fuel burning, cement manufacture and gas flaring for the period 1870–2009 (ref. 86) and RCPs for the period 2010–2100 (ref. 19). The global temperature change that occurred for the period 1961–2009 was calculated using S14FD[41] with the assumption that the global temperature change for the period 1986–2005, relative to 1850–1900, was 0.61 °C[87]. For the future period (2010–2100), the bias-corrected data of five GCMs[41] (see **Climate and other inputs** in the main text) were used. The overall relationship represented by the regression line in Supplementary Fig. S3 shows 1 °C and 5 °C warming from preindustrial levels for cumulative CO_2 emissions of 1,238 $GtCO_2$ and 7,652 $GtCO_2$, respectively.

Assumptions regarding future agricultural technologies and management

For all four crops, it was assumed that the future N application rates would gradually increase with income growth and scarcity of per capita agricultural area, with variations by country and SSP, and the increase in the N application rate would slow down or approach a plateau, as exemplified by China and India (Supplementary Fig. S20). These patterns are basically consistent with the assumption used in other works[55]. Interestingly, the difference in N application rates between SSP1 (rapid technological change) and SSP2 (intermediate technological change) was negligible, whereas the N application rate under SSP3 (slow technological change) was always lower than those under SSP1 and SSP2. However, caution is necessary because, in reality, the N application rate begins to gradually decrease after the plateau, reducing the environmental stress or producing high-quality grains at the expense of yield.

The knowledge stock of agricultural technologies assumed in this study rapidly increased with economic growth, with the exception of China for the period 2070–2100 (Supplementary Fig. S21). This tendency was consistent across SSPs. The differences in the knowledge stock across SSPs were more prominent than those in the N application rate. Although the knowledge stock in developing countries will rapidly increase in the future, the knowledge stock in these countries (except China and India) in 2100 will still be lower than that of the US in 2010.

References

50. World Bank. *World Development Indicators.*

http://data.worldbank.org/data-catalog/world-development-indicators (2016).
51. FAO. *FAOSTAT*. http://faostat3.fao.org/home/E (2016).
52. FAO. *Fertilizer and Plant Nutrition Bulletin: Fertilizer Use by Crop* (FAO, 2006).
53. IIASA. *SSP Database (Shared Socioeconomic Pathways) - Version 1.0*. https://tntcat.iiasa.ac.at/SspDb/dsd?Action=htmlpage&page=about (2016).
54. Jiang, L., & Li, Z. Urbanization and the change of fertilizer use sntensity for agricultural production in Henan province. *Sustain* **8**, 186 (2016).
55. FAO. *Fertilizer Requirements in 2015 and 2030* (FAO, 2000).
56. Conant, R. T., Berdanier, A. B., & Grace, P. R. Patterns and trends in nitrogen use and nitrogen recovery efficiency in world agriculture. *Glob. Biogeochem. Cycles* **27**, 558–566 (2013).
57. Cassman, K. G., Dobermann, A. R., & Walters, D. T. *Agroecosystems, Nitrogen-use Efficiency, and Nitrogen Management*. http://digitalcommons.unl.edu/agronomyfacpub/356 (2002).
58. IFPRI. *Agricultural Science and Technology Indicators*. https://www.asti.cgiar.org/ (2016).
59. Griliches, Z. and Mairesse, J. in *R&D, Patents, and Productivity* (eds Griliches, Z.) 339-374 (Univ. of Chicago Press, 1984).
60. Ito, J. Assessing the returns of R&D expenditures on postwar Japanese agricultural production. *Economic Review* **43**, 237–247 (1992).
61. Heisey, P., & Fuglie, K. *Economic Returns to Public Agricultural Research*. https://ssrn.com/abstract=1084926 (2007)
62. Huffman, W. E., & Evenson, R. E. Do Formula or Competitive Grant Funds Have Greater Impacts on State Agricultural Productivity? *Amer. J. Agric. Econ.* **88**, 783–798 (2006).
63. Pardey, P. G., & Alston, J. M. For Want of a Nail: The Case for Increased Agricultural R&D Spending (American Enterprise Institute, 2012)
64. Beintema, N., Stads, G.-J., Fuglie, K., & Heisey, P. *ASTI Global Assessment of Agricultural R&D Spending Developing Countries Accelerate Investment* (IFPRI, 2012).
65. Herdt, R. W. & Capule, C. *Adoption, Spread, and Production Impact of Modern Rice Varieties in Asia* (IRRI, 1983).
66. Jones, H. G. *Plants and Microclimate: A Quantitative Approach to Environmental Plant Physiology* (Cambridge Univ. Press, 2013).
67. Lassaletta, L., Billen, G., Grizzetti, B., Anglade, J. & Garnier, J. 50 year trends in nitrogen use efficiency of world cropping systems: the relationship between yield and nitrogen input to cropland. *Environ. Res. Lett.* **9**, 105011 (2014).
68. Monfreda, C., Ramankutty, N., & Foley, J. A. Farming the planet: 2. Geographic distribution of crop areas, yields, physiological types, and net primary production in the year 2000. *Glob. Biogeochem. Cycles* **22**, GB1022 (2008).
69. Tollenaar, M. & Wu, J. Yield improvement in temperate maize is attributable to greater stress tolerance. *Crop Sci.* **39**, 1597–1604 (1999).
70. Duvick, D. N. The contribution of breeding to yield advances in maize (*Zea mays L.*). Adv. Agron. **86**, 83–145 (2005).
71. Siebert, S. et al. A global data set of the extent of irrigated land from 1900 to 2005. *Hydrol. Earth Syst. Sci.* **19**, 1521–45 (2015).
72. Deryng, D., Sacks, W. J., Barford, C. C., & Ramankutty, N. Simulating the effects

of climate and agricultural management practices on global crop yield. *Glob. Biogeochem. Cycles* **25**, GB2006 (2011).

73. Neitsch, S. L., Arnold, J. G., Kiniry, J. R., & Williams, J. R. *Soil and Water Assessment Tool Theoretical Documentation (Version 2005)* (USDA, 2005).
74. Osborne, T. et al. JULES-crop: a parametrisation of crops in the Joint UK Land Environment Simulator. *Geosci. Model Dev.* **8**, 1139–1155 (2015).
75. Sawano, S. et al. Effects of nitrogen input and climate trends on provincial rice yields in China between 1961 and 2003: quantitative evaluation using a crop model. *Paddy Water Environ.* **13**, 529–543 (2015).
76. Rosenzweig, C., Tubiello, F. N., Goldberg, R., Mills, E., & Bloomfield, J. Increased crop damage in the US from excess precipitation under climate change. *Glob. Environmen. Change* **12**, 197–202 (2002).
77. Wang, R., Bowling, L. C., & Cherkauer, K. A. Estimation of the effects of climate variability on crop yield in the Midwest USA. *Agric. For. Meteorol.* **216**, 141–156 (2016).
78. Trnka, M. et al. Simple snow cover model for agrometeorological applications. *Agric. For. Meteorol.* **150**, 7–8 (2010).
79. Huang, J., van den Dool, H. M., & Georgarakos, K. P. Analysis of model-calculated soil moisture over the United States (1931–1993) and applications to long-range temperature forecasts. *J. Clim.* **9**, 1350–1362 (1996).
80. Waha, K., van Bussel, L. G. J., Müller, C., & Bondeau, A. Climate-driven simulation of global crop sowing dates. *Glob. Ecol. Biogeogr.* **21**, 247–259 (2012).
81. Licker, R. et al. Mind the gap: how do climate and agricultural management explain the ‘yield gap’ of croplands around the world? *Glob. Ecol. Biogeogr.* **19**, 769–782 (2010).
82. David, C. C. & Otsuka, K. *Modern Rice Technology and Income Distribution in Asia* (IRRI, 1994).
83. Edgerton, M. D. Increasing crop productivity to meet global needs for feed, food, and fuel. *Plant Physiol.* **149**, 7–13 (2009).
84. Long, S. P., Zhu, X.-G., Naidu, S. L., & Ort, D. R. Can improvement in photosynthesis increase crop yields? *Plant, Cell & Environ.* **29**, 315–330 (2006).
85. Müller, C. *et al.* Global Gridded Crop Model evaluation: benchmarking, skills, deficiencies and implications. *Geosci. Model Dev.*, **10**, 1403–1422 (2017).
86. Boden, T. A., Marland, G., & Andres, R. J. *Global, Regional, and National Fossil-Fuel CO_2 Emissions* (CDIAC, 2016).
87. IPCC. in *Climate Change 2013: The Physical Science Basis*. (eds Stocker, T. F. et al.) 3–29 (Cambridge Univ. Press, 2013).
88. Bouman, B. A. M. et al. *ORYZA2000: modeling lowland rice* (IRRI, 2001).

Supplementary Table S1. The anticipated global decadal mean relative yields of four crops in 2100 based on those of 2010. Ensemble mean values (and the minimum and maximum shown in the parentheses), derived using the five ensemble members of the climate data, are presented.

RCP and global temperature change	SSP	Global mean yield increases in 2100 relative to 2010			
		Maize	Soybean	Rice	Wheat
RCP2.6 (+1.8 °C)	1	1.27 (1.19,1.33)	1.56 (1.38,1.64)	1.40 (1.38,1.41)	1.61 (1.53,1.71)
	2	1.26 (1.18,1.32)	1.51 (1.34,1.58)	1.39 (1.38,1.41)	1.59 (1.52,1.69)
	3	1.19 (1.11,1.25)	1.31 (1.17,1.36)	1.35 (1.34,1.37)	1.44 (1.37,1.53)
RCP4.5 (+2.7 °C)	1	1.11 (0.97,1.30)	1.37 (1.10,1.71)	1.47 (1.40,1.54)	1.59 (1.53,1.64)
	2	1.11 (0.96,1.29)	1.32 (1.08,1.64)	1.47 (1.40,1.53)	1.57 (1.51,1.62)
	3	1.05 (0.90,1.23)	1.15 (0.95,1.41)	1.43 (1.36,1.49)	1.40 (1.35,1.47)
RCP6.0 (+3.2 °C)	1	1.03 (0.82,1.20)	1.32 (0.97,1.54)	1.55 (1.47,1.59)	1.67 (1.61,1.71)
	2	1.02 (0.81,1.19)	1.27 (0.95,1.48)	1.55 (1.46,1.58)	1.65 (1.59,1.69)
	3	0.96 (0.76,1.13)	1.11 (0.84,1.27)	1.50 (1.42,1.54)	1.48 (1.43,1.52)
RCP8.5 (+4.9 °C)	1	0.61 (0.39,0.94)	0.85 (0.55,1.27)	1.49 (1.22,1.64)	1.59 (1.45,1.78)
	2	0.61 (0.39,0.94)	0.83 (0.54,1.23)	1.48 (1.22,1.63)	1.57 (1.43,1.76)
	3	0.58 (0.36,0.89)	0.73 (0.49,1.06)	1.45 (1.19,1.59)	1.43 (1.30,1.62)
noCC (+0.6 °C)	1	1.39 (1.37,1.40)	1.65 (1.61,1.67)	1.29 (1.27,1.33)	1.56 (1.51,1.60)
	2	1.38 (1.35,1.39)	1.59 (1.56,1.62)	1.28 (1.26,1.32)	1.54 (1.50,1.58)
	3	1.30 (1.28,1.31)	1.38 (1.35,1.40)	1.25 (1.23,1.28)	1.40 (1.35,1.43)

Supplementary Table S1. (continued)

RCP and global temperature change	SSP	Maize	Soybean	Rice	Wheat
+1.5 °C	1	1.30	1.59	1.37	1.60
	2	1.29	1.53	1.36	1.58
	3	1.22	1.33	1.33	1.43
+2.0 °C	1	1.24	1.52	1.42	1.61
	2	1.23	1.47	1.41	1.59
	3	1.16	1.27	1.37	1.43

Supplementary Table S2. Regression coefficients in equation (1) describing the relationship between N fertilizer consumption, per capita GDP and per capita agricultural area. Data for selected major crop-producing countries are presented. The asterisks indicate the significance: ***, 0.1%; **, 1%; and *, 5%.

Country	Adj-R^2	Coefficients		
		a (per capita GDP)	*b* (per capita agricultural area)	*c* (intercept)
United States	0.952	61.307 ***	77.092 ***	-652.471 ***
China	0.981	17.518 ***	-28.377 *	-103.444 ***
India	0.996	32.393 ***	-43.656 ***	-228.497 ***
Brazil	0.880	0.841	-17.522 ***	6.110
Argentina	0.737	8.111 ***	-3.429 **	-64.967 **
Indonesia	0.988	26.976 ***	-9.645 *	-176.742 ***
Bangladesh	0.992	47.150 ***	-99.356 ***	-450.233 ***
France	0.974	229.22 ***	412.81 ***	-2014.95 ***

Supplementary Table S3. List of the GCMs and modeling groups obtained from the CMIP5 multimodel ensemble dataset[43] for this study.

GCM name	Modeling group
GFDL-ESM2M	NOAA Geophysical Fluid Dynamics Laboratory
IPSL-CM5A-LR	Institut Pierre-Simon Laplace
HadGEM2-ES	Met Office Hadley Centre
MIROC-ESM-CHEM	Japan Agency for Marine-Earth Science and Technology, Atmosphere and Ocean Research Institute (The University of Tokyo), and National Institute for Environmental Studies
NorESM1-M	Norwegian Climate Centre

Supplementary Table S4. Coefficient values used in the crop model.

Variable	Eq(s)	Maize	Soybean	Rice	Wheat	Reference(s)
Development						
a (-)	14	I[1]: 3466<br>R[2]: 3296	I: 3719<br>R: 2809	I: 163689<br>R:163689	I: 7404<br>R:10340	This work (Supplementary Fig. S16)
b (-)	14	I: 7546<br>R: 7028	I: 9892<br>R:7144	I: 491572<br>R:491572	I:14297<br>R:12343	
T_b (°C)	15, 29	8	10	8	0	refs. 72, 73 and 88
T_u (°C)	15, 28	30	30	37	26	
T_o (°C)	15, 28, 29	25	25	25	18	
Growth						
$fr_{GDD,\ sen}$ (-)	16	0.6	0.6	0.6	0.6	ref. 73
LAI_{max} ($m^2\ m^{-2}$)	17	3.	3.	5.	4.	
l_1 (-)	18	3.055	3.055	8.410	3.055	
l_2 (-)	18	13.385	13.385	16.731	13.385	
h_{max} (m)	20	2.5	0.8	0.8	0.9	
Yield formation						
p_1 (-)	23	12.915	8.584	20.397	17.196	ref. 74
p_2 (-)	23	25.433	27.900	35.517	29.156	
$fr_{GDD,\ ant}$ (-)	23	0.482	0.301	0.552	0.562	Unpublished work[3]
k (-)	25	0.65	0.65	0.65	0.65	ref. 73
r_1 (-)	26	5.8000	6.6399	6.8372	6.1836	
r_2 (-)	26	-0.0014	-0.0008	-0.0007	-0.0007	
Stresses (with adjustment based on knowledge stock)[4]						
$Napp_o$ (kg N $ha^{-1}\ yr^{-1}$)	27	210	50	210	210	This work (Supplementary Fig. S14)
$Napp_{min}$ (kg N $ha^{-1}\ yr^{-1}$)	27	-550	-220	-650	-500	
f_{Ndef} (-)	32	-0.0264	-0.0264	-0.0138	-0.0244	
g_{Ndef} (-)	32	10.6629	13.7706	12.2711	9.8026	
f_{heat} (-)	-	-0.0025	-0.0026	-0.0035	-0.0025	
g_{heat} (-)	-	0.1689	0.2316	0.0449	0.2056	
f_{cold} (-)	-	-0.0025	-0.0025	-0.0025	-0.0025	
g_{cold} (-)	-	0.3190	0.4297	0.4862	0.4049	
f_{Wdef} (-)	-	-0.0025	-0.0005	-0.0014	-0.0026	
g_{Wdef} (-)	-	0.1496	0.1183	0.0253	0.3993	
f_{Wexs} (-)	-	-0.00025	-0.00026	-0.00026	-0.00021	
g_{Wexs} (-)	-	0.04579	0.04174	0.02046	0.01563	

Supplementary Table S4. (continued)

Variable	Eq(s)	Maize	Soybean	Rice	Wheat	Reference(s)
Stresses (default)						
α_{Ndef}	Supplementary Methods [5]	8.	8.	8.	8.	ref. 73 [6]
α_{heat}		0.1054	0.1054	0.1054	0.1054	ref. 73
α_{cold}		0.1054	0.1054	0.1054	0.1054	
α_{Wdef}		0.5	0.5	0.5	0.5	ref. 77 [7]
α_{Wexs}		0.01	0.01	0.01	0.01	

[1] Irrigated cropping system.
[2] Rainfed cropping system.
[3] An unpublished work conducted by T.I. that uses the collected field experimental data across the major crop-producing countries.
[4] The lower and upper limits of the curvature factor value are: N deficit, (0.25, 20); heat, (0.02, 5); cold, (0.1, 1.); water deficit, (0.01, 0.8); and water excess, (0.002, 0.1).
[5] See **The use of improved technologies and management systems** in **Supplementary Methods**.
[6] For maize, rice and wheat, the default curvature factor values for N deficit were determined to follow the shape of the stress function presented in the literature. The default values for soybean were determined with the assumption that N stress is roughly half that of other crops.
[7] The default curvature factor values for water deficit and water excess were determined to follow the shape of the stress functions presented in the literature.

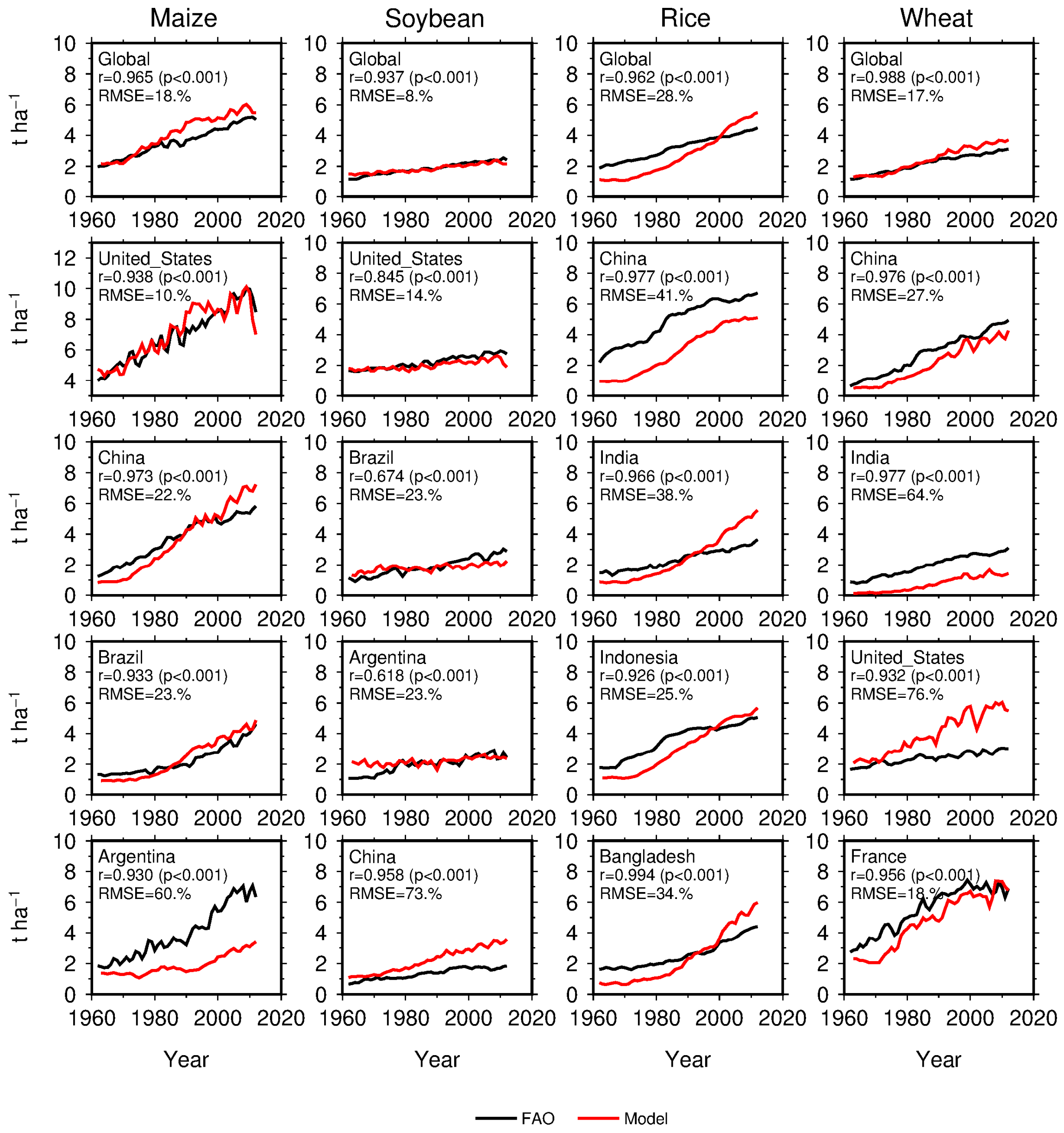

Supplementary Figure S1. Global and country mean yields of four crops for selected major crop-producing countries for the period 1961–2012, calculated using the modeled and FAO-reported data. The correlation (r), p-value (p) and root-mean-square error relative to the mean reported yield for the period 1961–2012 (RMSE) are also presented.

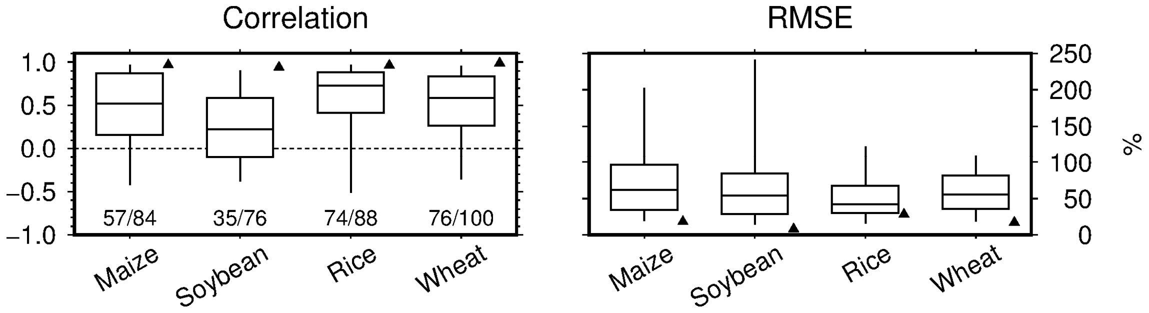

Supplementary Figure S2. Summary of the comparisons between the modeled and reported yields for four crops. A box plot in the left panel summarizes the correlations calculated between the modeled and reported data for the period 1961–2012 (the sample size varies by country but is greater than 20) across countries; the vertical line of a box plot indicates the 90% probability interval; a box indicates the 50% probability interval; a horizontal line in a box indicates the median; a triangle on the right-hand side of a box plot indicates the correlation for the global mean yield; and the numbers below a box plot indicate the number of countries examined and the number of countries with a significant correlation. The box plot in the right panel summarizes the root-mean-square error relative to the mean reported yield for the period 1961–2012 (RMSE), as in the left panel.

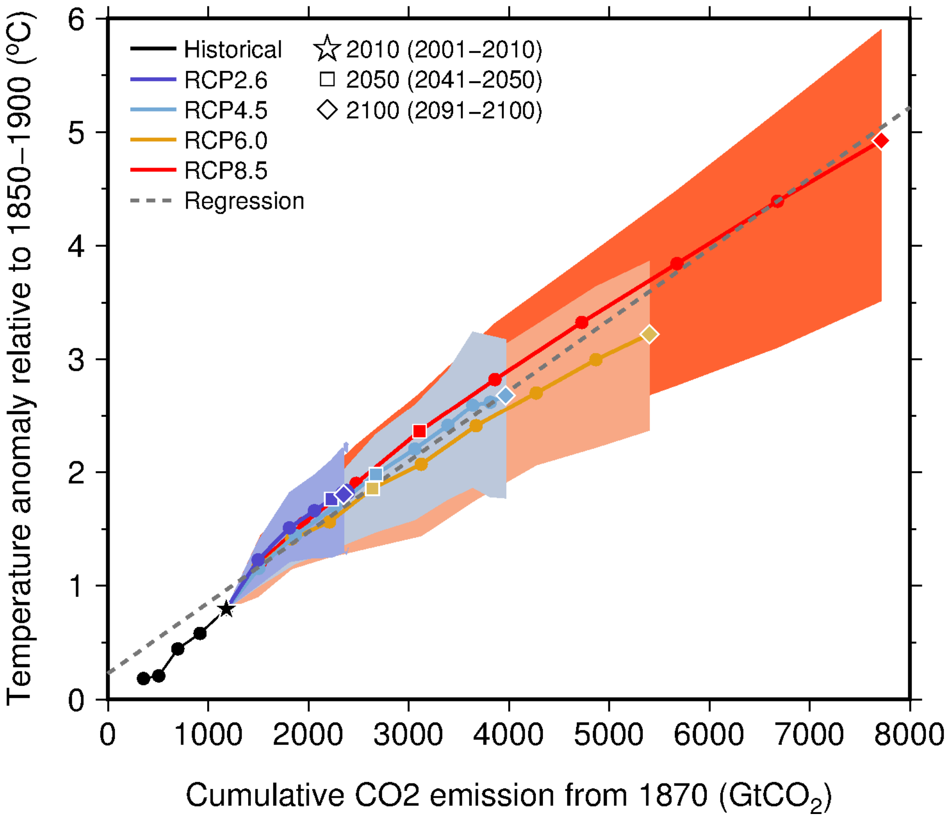

Supplementary Figure S3. Global decadal mean surface temperature anomaly relative to preindustrial time period (1850–1900) as a function of cumulative total global CO_2 emissions from 1870. Some decadal means are labeled for clarity (e.g., 2100 indicates the decade 2091–2100). Colored solid lines with dots indicate the future projection represented by the ensemble mean for each RCP (calculated using 5 GCMs). The colored shaded area indicates the ensemble spread (from minimum to maximum) for each RCP. The black solid line indicates the historical past calculated by using the S14FD meteorological forcing dataset. The gray dashed line indicates a regression line fitted to the data from the historical past and four RCPs. See **Global temperature change and cumulative CO_2 emission** in **Supplementary Note** for more details.

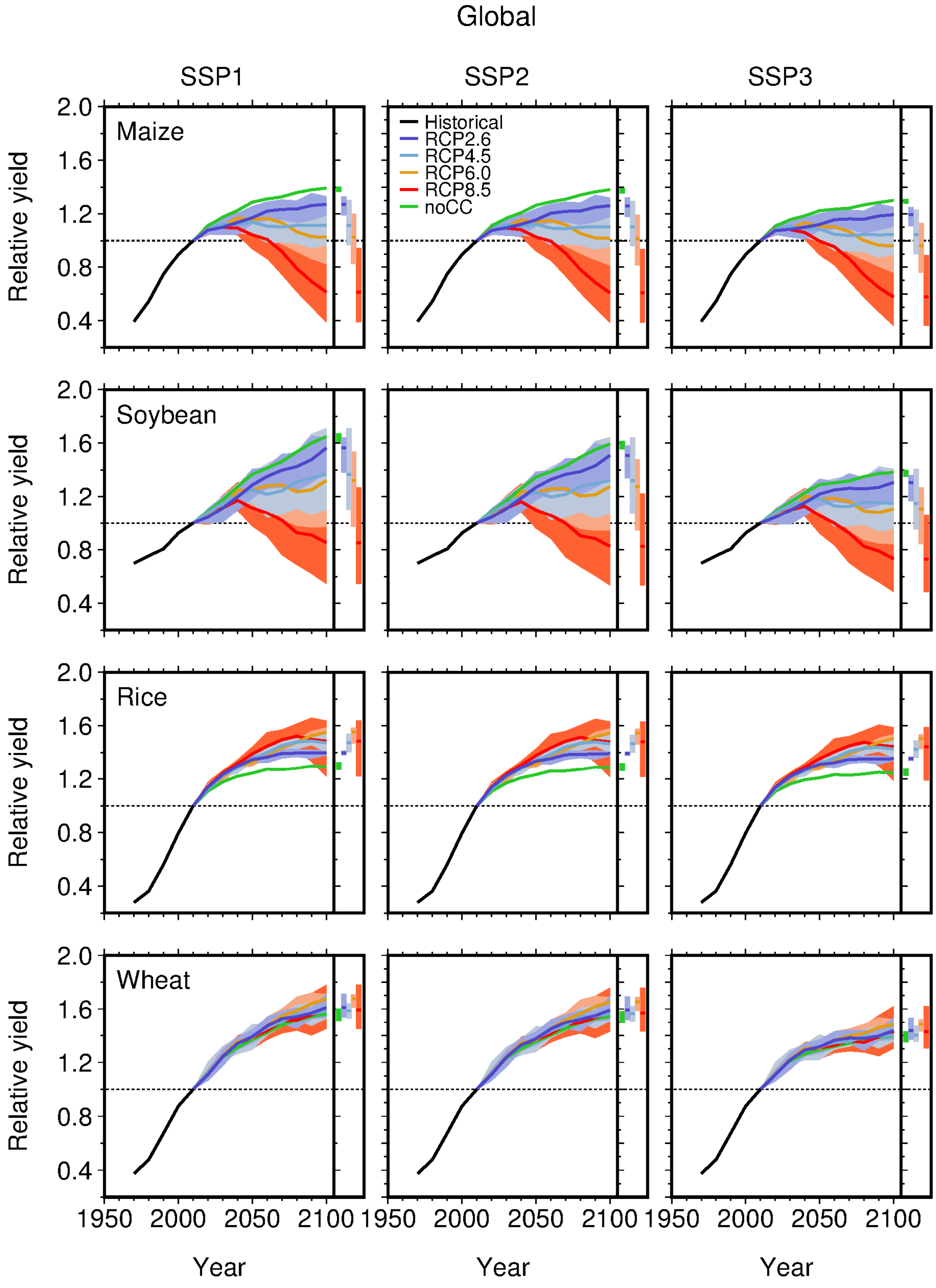

Supplementary Figure S4. Modeled global mean yield growth of maize, soybean, rice and wheat as a function of time. The crop yield is scaled so that the mean yield in 2010 (the average for the period 2001–2010) is equal to one. Solid black lines indicate the historical run (1961–2009). Colored solid lines indicate the ensemble mean for each RCP under a given SSP, calculated using 5 members (5 GCMs) (2010–2100), derived from the future run. The colored shaded area indicates the ensemble spread (from minimum to maximum) across the members. A bar shows the ensemble mean and spread for 2100 (the average for the period 2091–2100) for each RCP. Data derived from the no-climate-change (noCC) run are also presented as a reference.

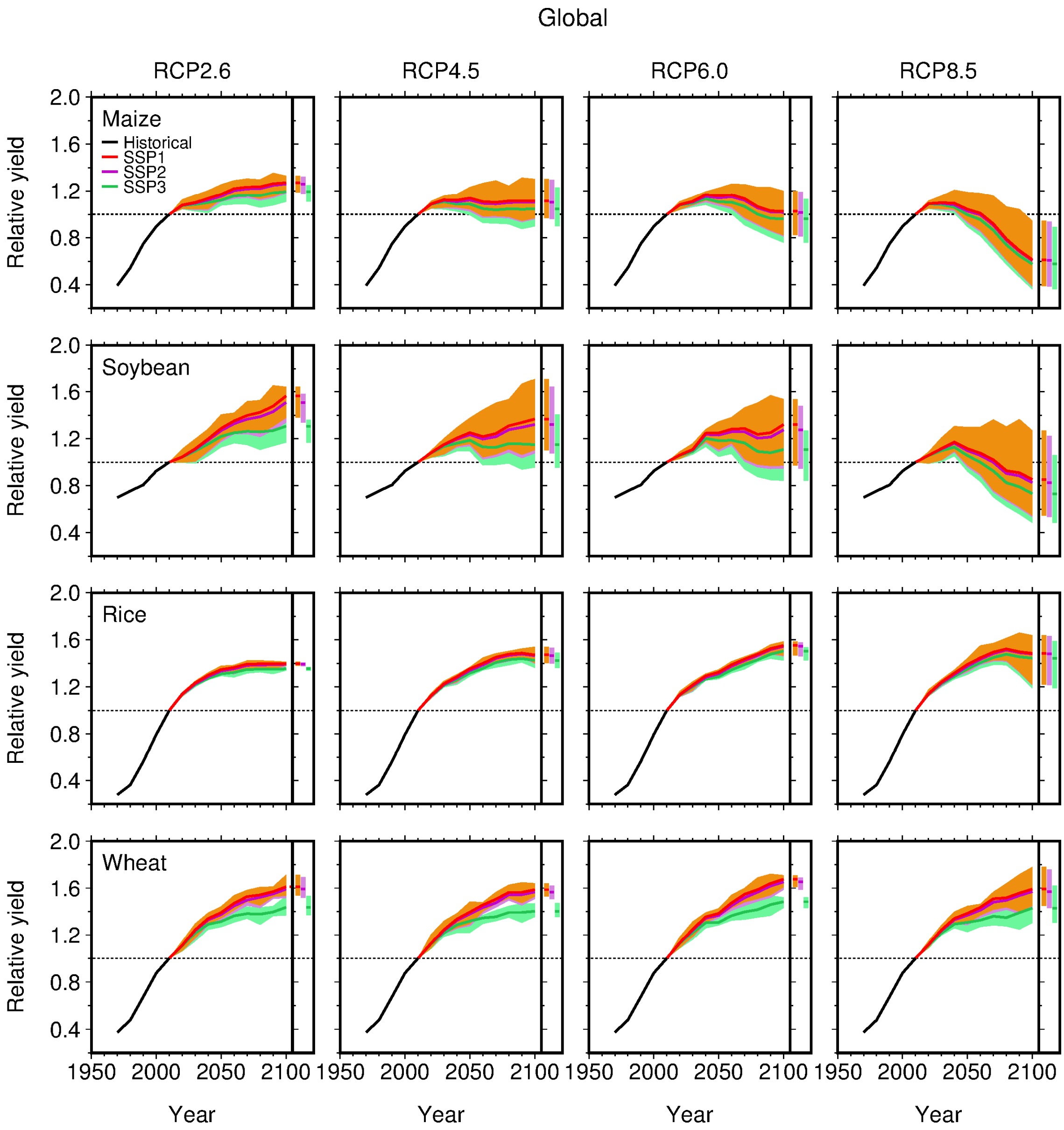

Supplementary Figure S5. Modeled global mean yield growth of maize, soybean, rice and wheat as a function of time. The crop yield is scaled so that the mean yield in 2010 (the average for the period 2001–2010) is equal to one. Solid black lines indicate the historical run (1961–2009). Colored solid lines indicate the ensemble mean for each SSP under a given RCP, calculated using 5 members (5 GCMs) (2010–2100). The colored shaded area indicates the ensemble spread (from minimum to maximum) across the members. A bar shows the ensemble spread for 2100 (the average for the period 2091–2100) for each SSP.

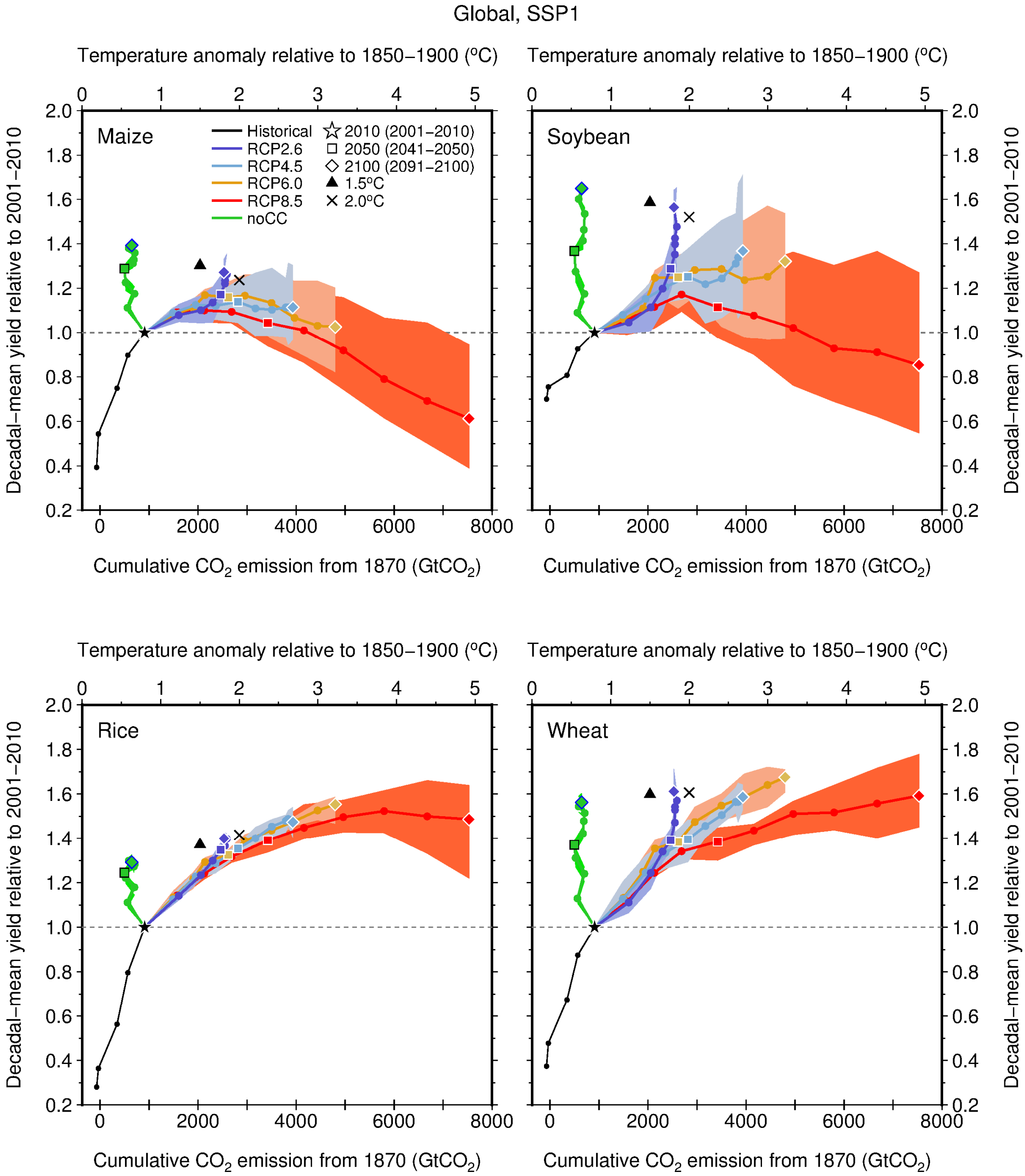

Supplementary Figure S6. Same as Fig. 2, but SSP1 (rapid technological change) was assumed.

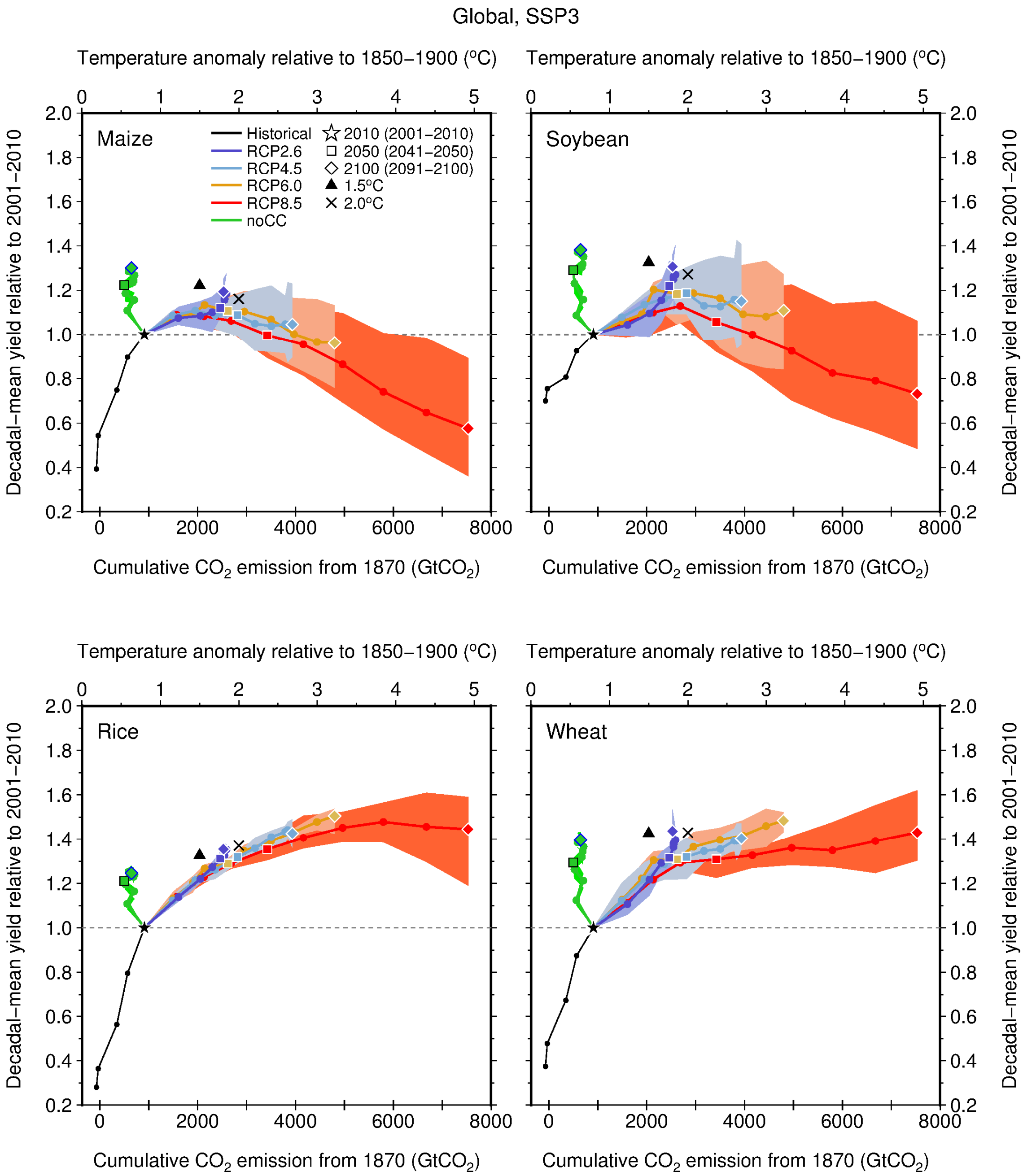

Supplementary Figure S7. Same as Fig. 2, but SSP3 (slow technological change) was assumed.

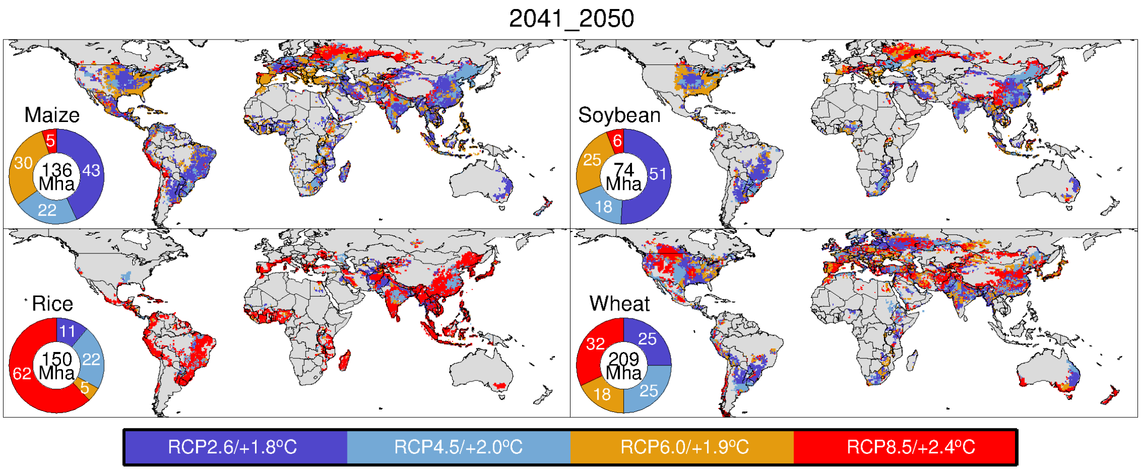

Supplementary Figure S8. Same as Fig. 3, but for 2050 (the average for the period 2041–2050). The maps presented here were created with the Generic Mapping Tools (GMT)[49] version 4.5.12 (https://www.soest.hawaii.edu/gmt/) using data sources described in the main text.

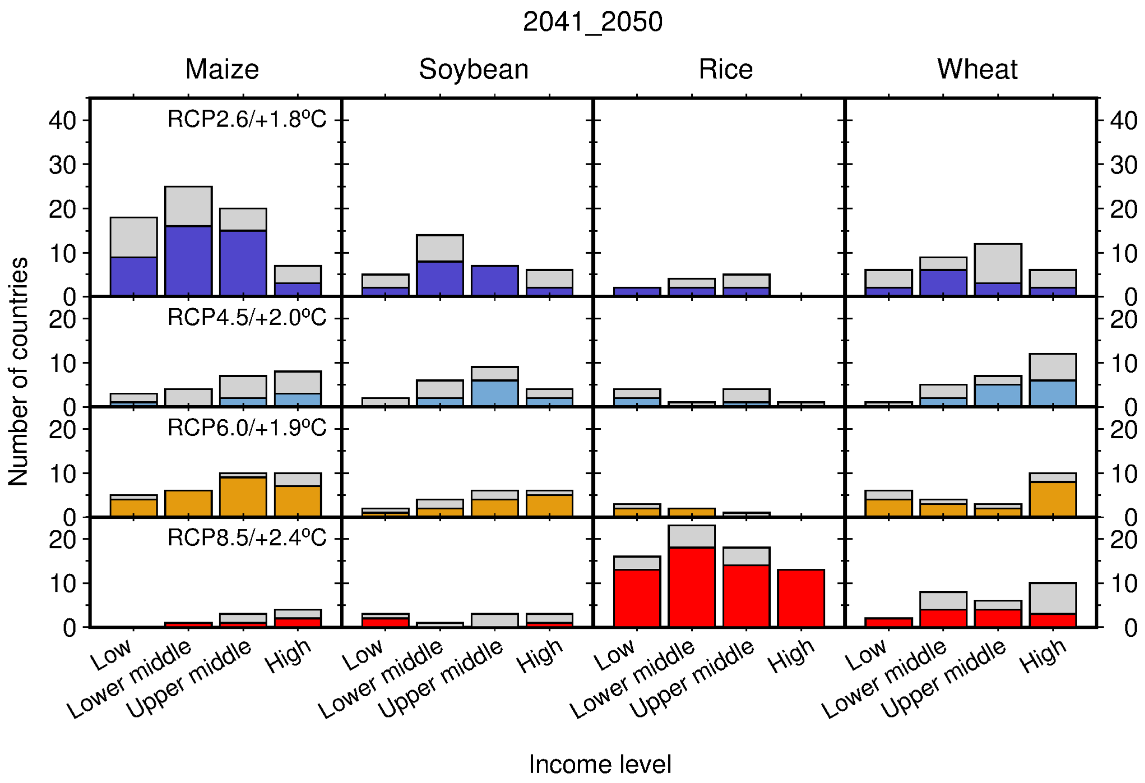

Supplementary Figure S9. Same as Fig. 4, but for 2050 (the average for the period 2041–2050).

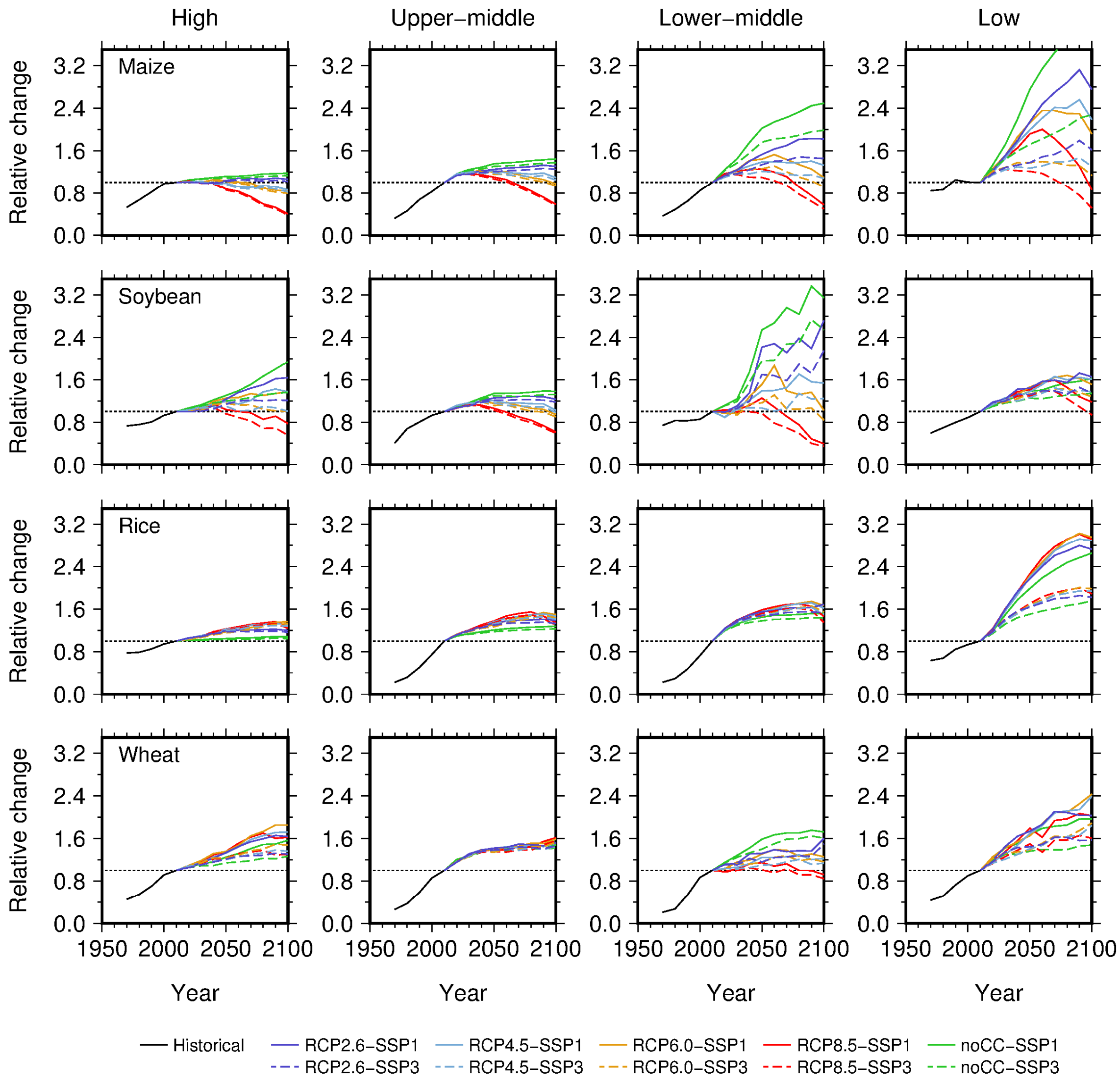

Supplementary Figure S10. Modeled mean yield growth of maize, soybean, rice and wheat over all countries by income level. The crop yield is scaled so that the mean yield in 2010 (the average for the period 2001–2010) is equal to one. Solid black lines indicate the historical run (1961–2009). Colored solid and dashed lines indicate the ensemble mean for each combination of RCP and SSP, calculated using five GCMs (2010–2100).

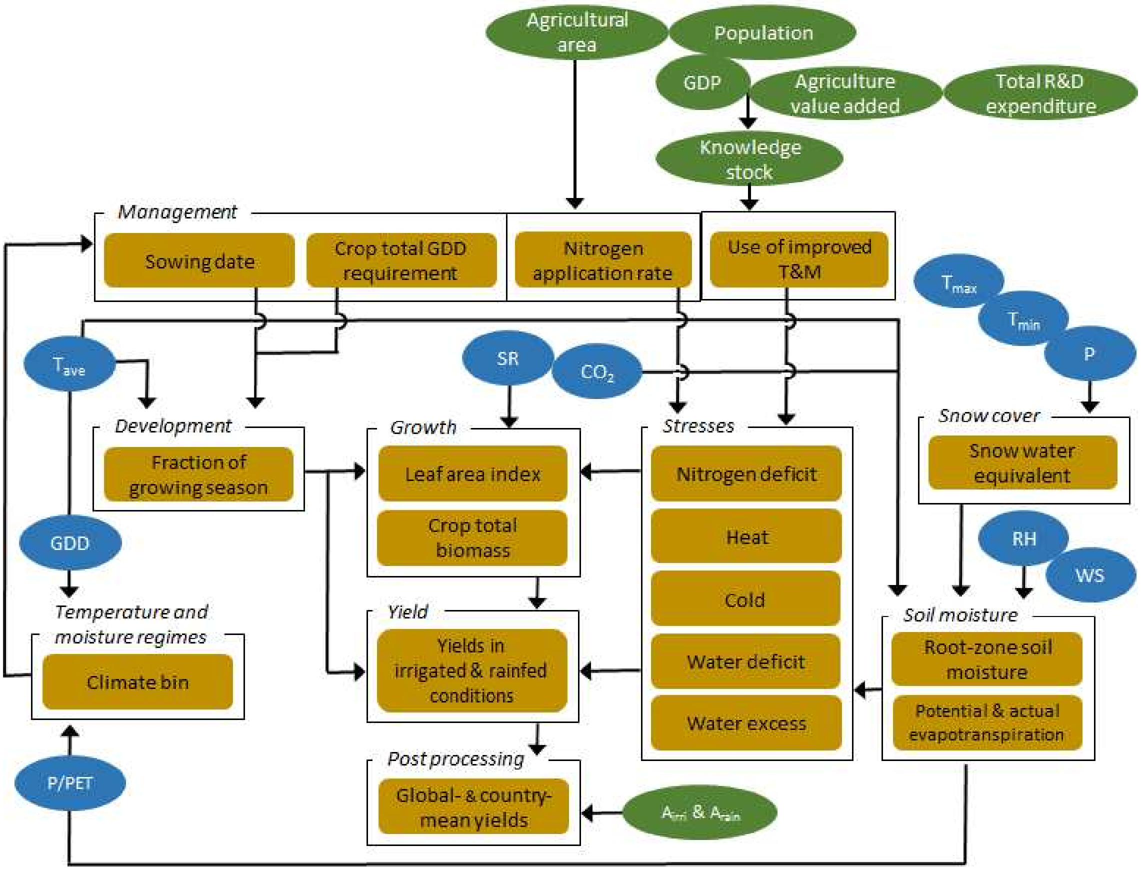

Supplementary Figure S11. Schematic illustrating the CYGMA global gridded crop model. A blue ellipse indicates a physical variable: T_{ave}, T_{max} and T_{min}, daily mean, maximum and minimum temperatures, respectively; P, precipitation; SR, downward shortwave radiation; RH, relative humidity, WS, wind speed; CO_2, atmospheric carbon dioxide concentration; GDD, growing-degree days and P/PET, aridity index. A green ellipse indicates a socioeconomic variable: R&D, research and development; T&M, technologies and management; and A_{irri} and A_{rain}, irrigated and rainfed areas, respectively.

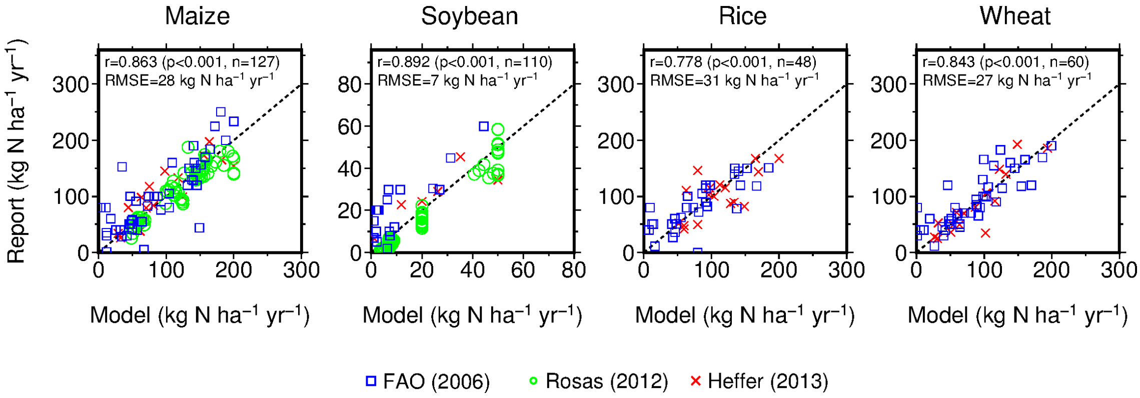

Supplementary Figure S12. Modeled and reported N application rates by crop for selected countries and years. The data period and number of countries vary by data source: ref. 52 or FAO (2006), 27–65 countries for the period from 1997–2004; ref. 24 or Rosas (2012), 4–5 countries for the period from 1990–2010; and ref. 40 or Heffer (2013), 29 countries for 2010 or 2010/11.

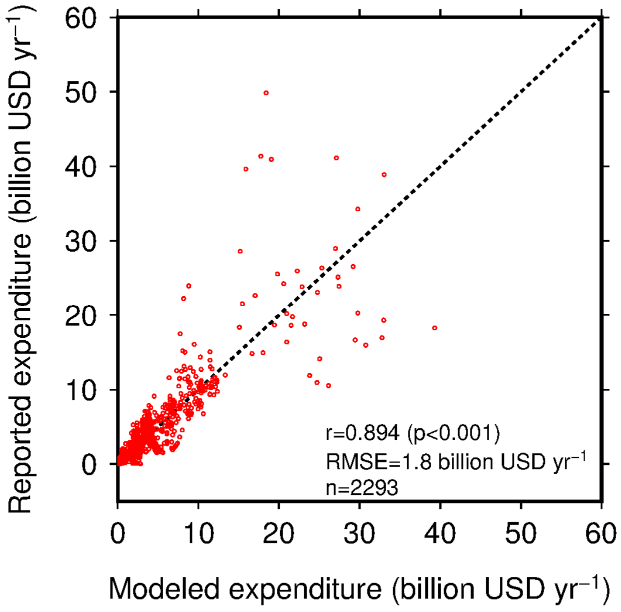

Supplementary Figure S13. Modeled and IFPRI-reported agricultural R&D expenditure for selected countries during the period 1980–2012.

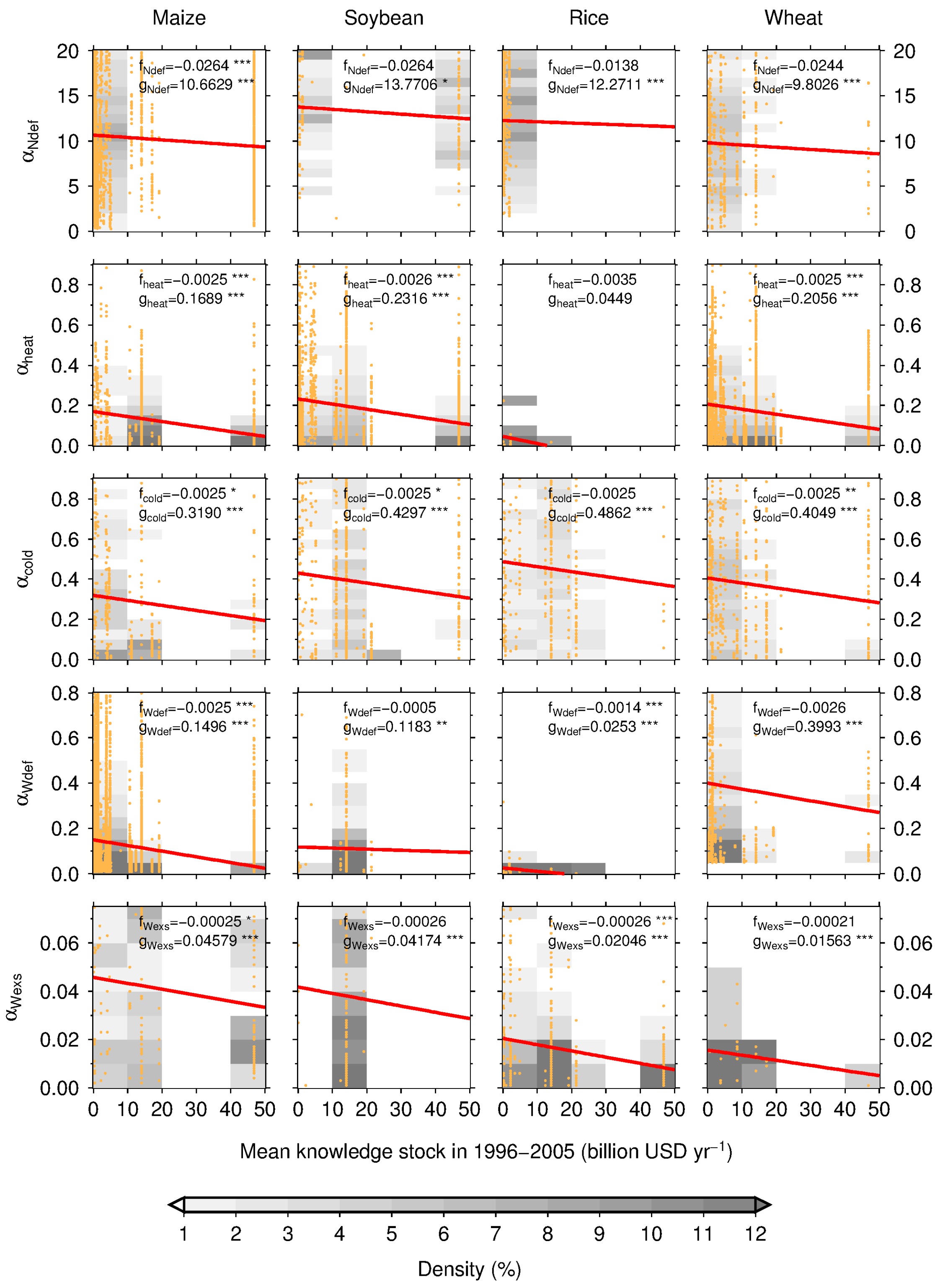

Supplementary Figure S14. Relationships between the grid-cell curvature factor value and mean knowledge stock in 1996–2005 by stress type and crop. Orange dots indicate grid-cell data; the gray shaded area indicates the density of data; and the red line indicates the regression line fitted to the data. The asterisks indicate the significance: ***, 0.1%; **, 1%; and *, 5%.

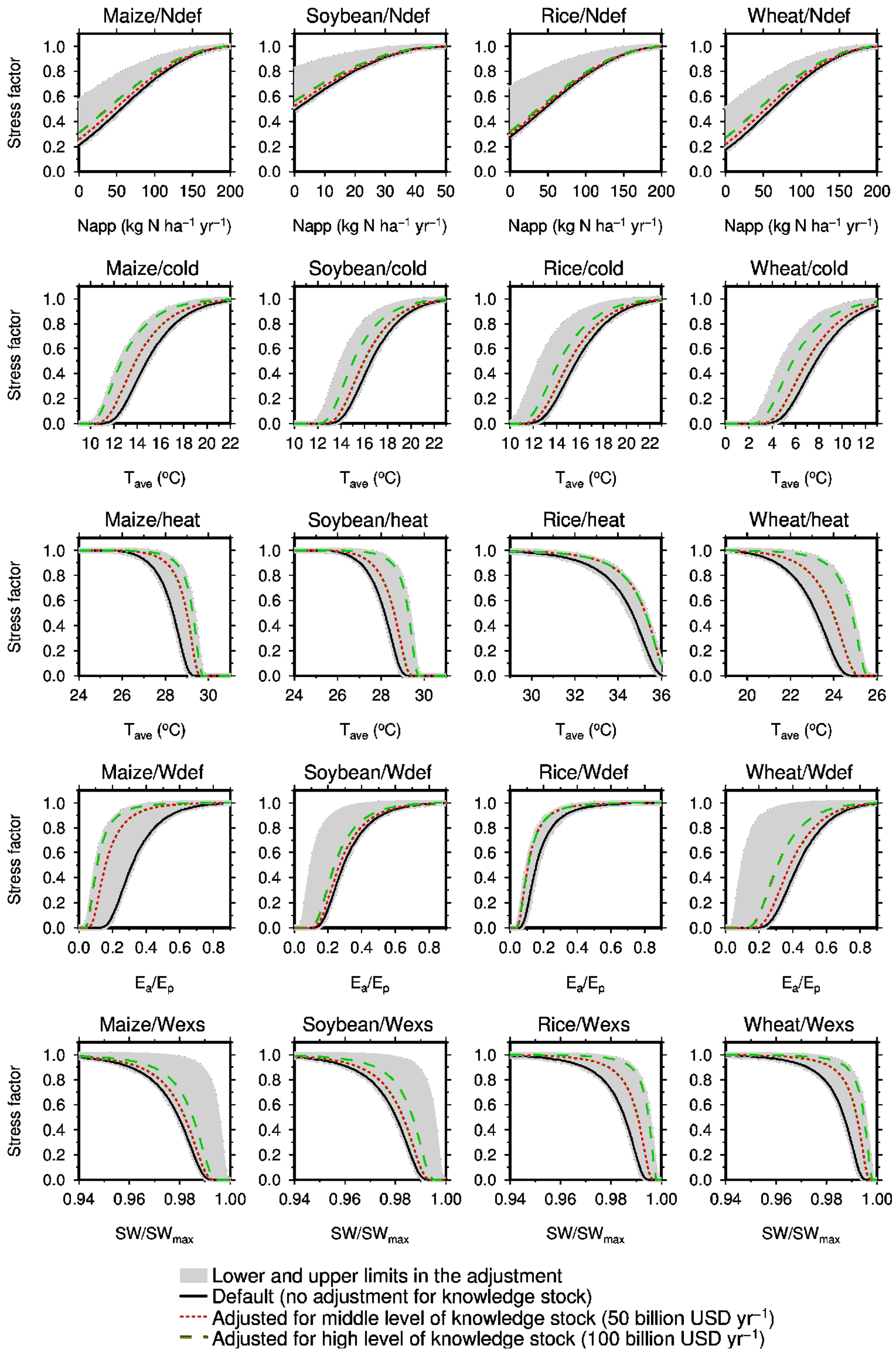

Supplementary Figure S15. Models of five different stress factors for four crops associated with N deficit (Ndef), cold, heat, water deficit (W_{def}) and water excess (W_{exs}). T_{ave} indicates the daily mean temperature; E_a/E_p indicates the ratio of actual-potential evapotranspiration rates; and SW/SW_{max} indicates the ratio of root-zone soil moisture relative to the plant-extractable water capacity of soil. The stress factor ranges from 0 under the severely stressed condition to 1 under the optimal condition.

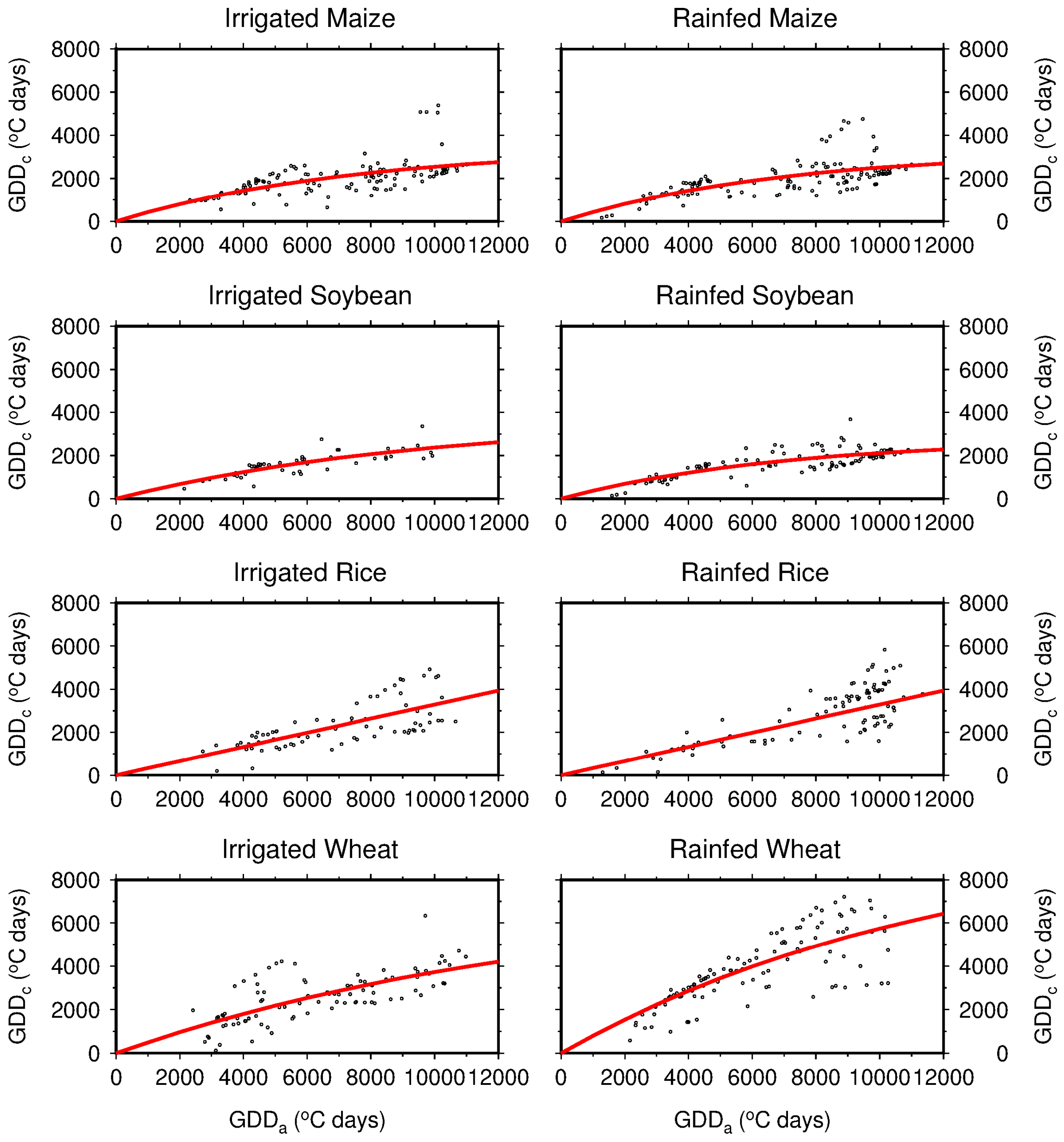

Supplementary Figure S16. Relationships between the mean annual GDD for the period 1996–2005 (GDD_a) and the crop total GDD requirement in 2000 (GDD_c) for four crops. A dot indicates a data value in a grid cell with the largest irrigated (or rainfed) area in a country. A red line indicates a fitted nonlinear function, $GDD_c=a*(1–\exp[-GDD_a/b])$. See Supplementary Table S4 for the values of *a* and *b*.

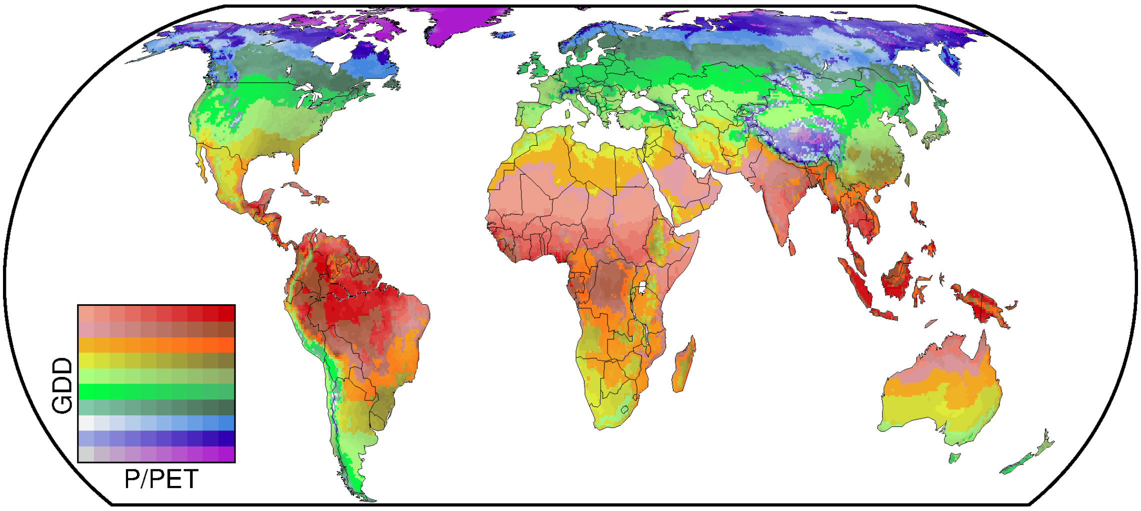

Supplementary Figure S17. An example of the climate bins calculated using the S14FD meteorological forcing dataset for the period 1996–2005. The maps presented here were created with the Generic Mapping Tools (GMT)[49] version 4.5.12 (https://www.soest.hawaii.edu/gmt/) using data sources described in **Supplementary Note**.

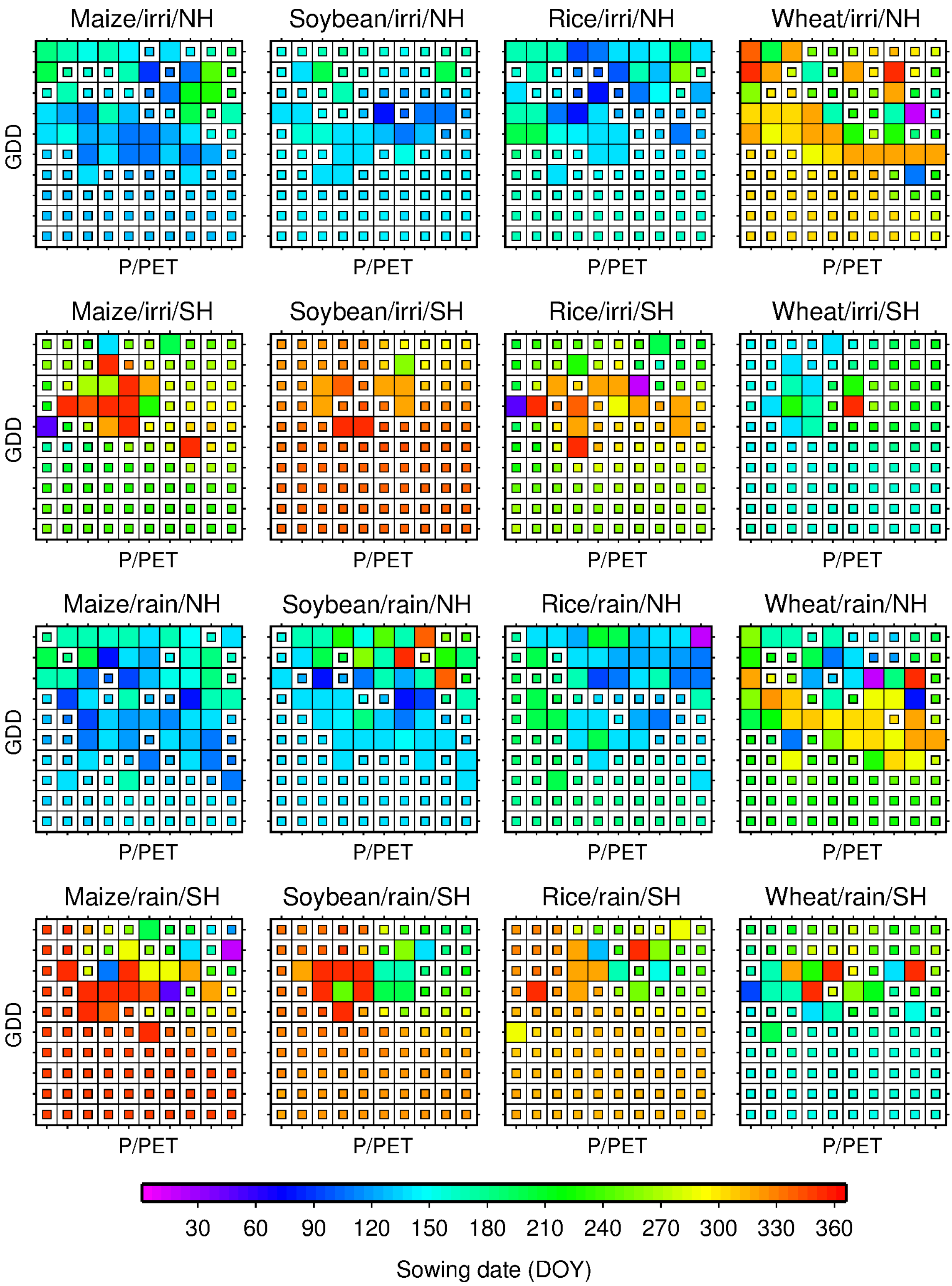

Supplementary Figure S18. Sowing dates of four crops specific to the climate bins, cropping systems (irrigated or rainfed) and hemispheres (NH, northern hemisphere; and SH, southern hemisphere) used in the model. A large tile indicates that a data value was calculated using MIRCA2000. A small tile indicates that a data value was extrapolated from the four neighboring climate bins.

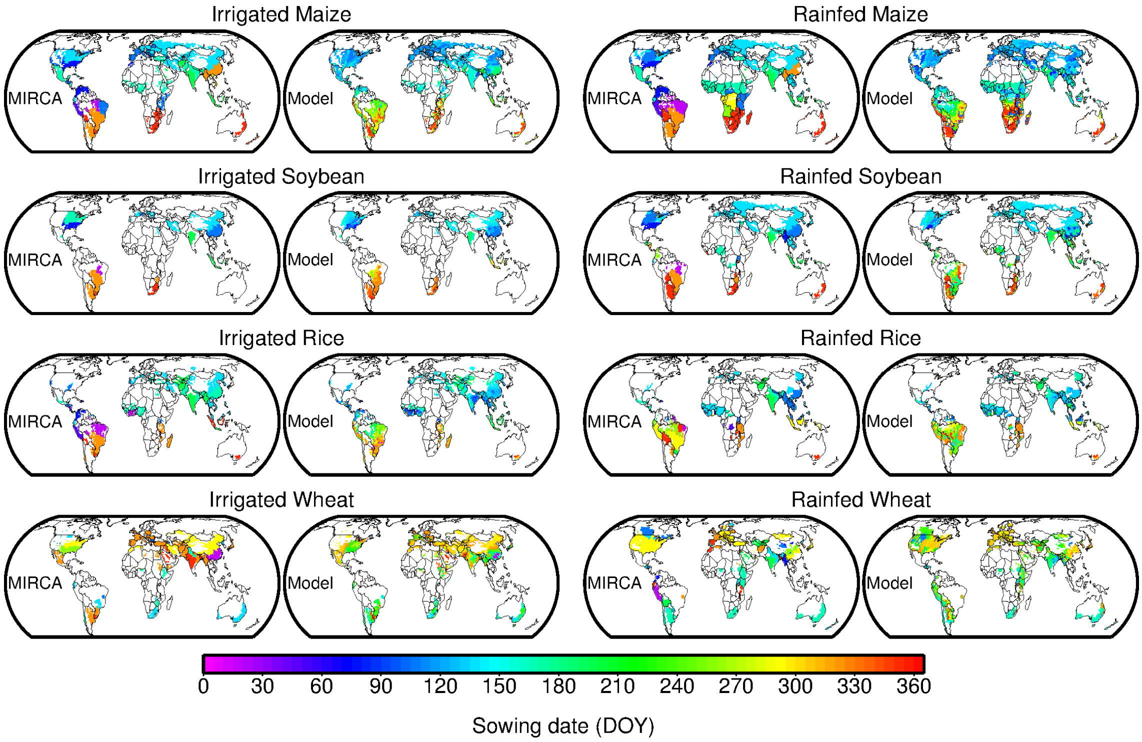

Supplementary Figure S19. MIRCA2000-reported and modeled sowing dates of four crops for irrigated and rainfed cropping systems in 2000. The maps presented here were created with the Generic Mapping Tools (GMT)[49] version 4.5.12 (https://www.soest.hawaii.edu/gmt/) using data sources described in **Supplementary Note**.

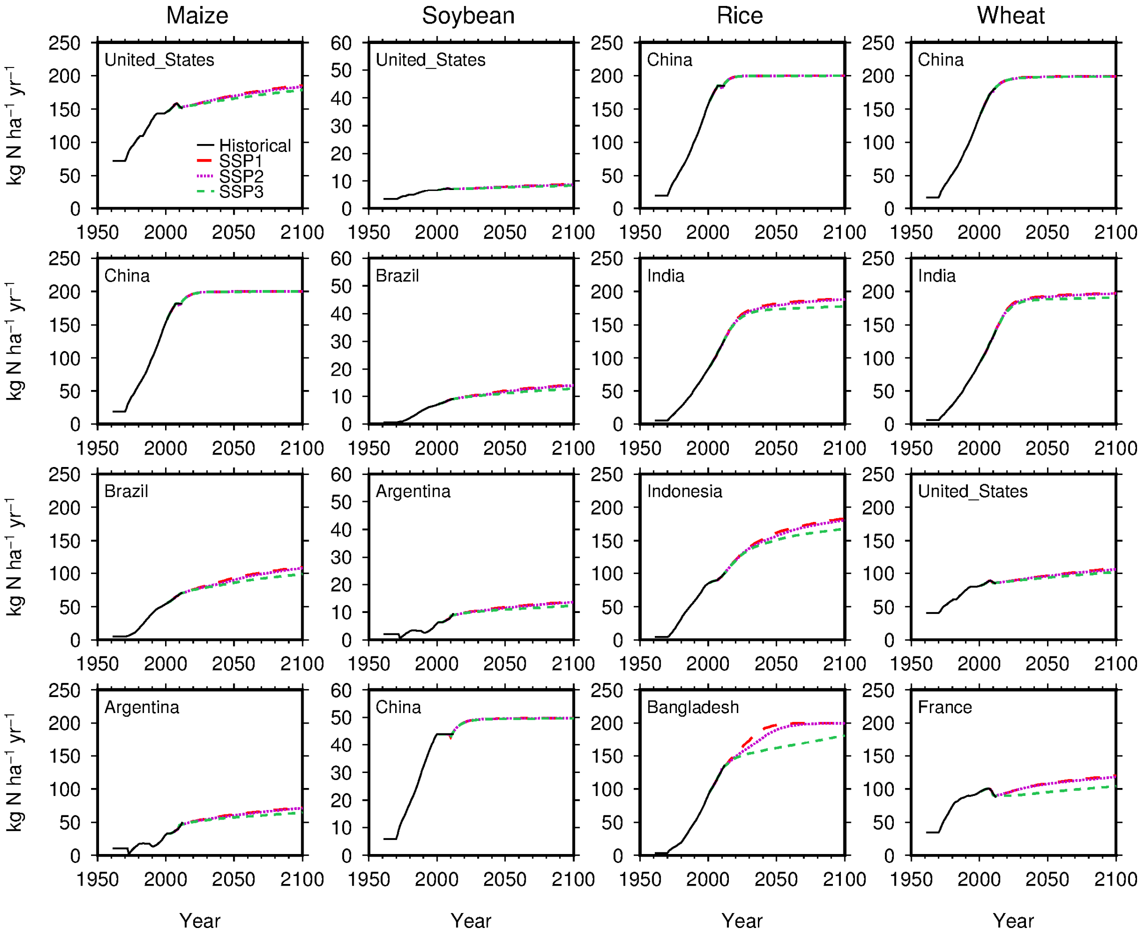

Supplementary Figure S20. Assumptions regarding the N application rates of the four crops for selected major crop-producing countries in the historical and future periods.

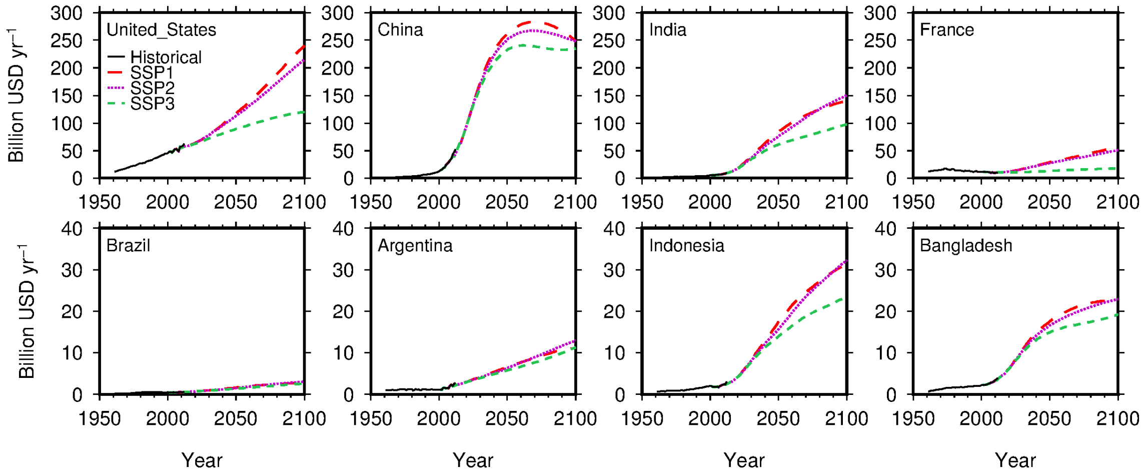

Supplementary Figure S21. Assumptions regarding the knowledge stock of agricultural technologies for selected major crop-producing countries in the historical and future periods.
